# Zoledronic acid-encapsulating self-assembling nanoparticles and doxorubicin: a combinatorial approach to overcome simultaneously chemoresistance and immunoresistance in breast tumors

**DOI:** 10.18632/oncotarget.8012

**Published:** 2016-03-09

**Authors:** Joanna Kopecka, Stefania Porto, Sara Lusa, Elena Gazzano, Giuseppina Salzano, Martha Leonor Pinzòn-Daza, Antonio Giordano, Vincenzo Desiderio, Dario Ghigo, Giuseppe De Rosa, Michele Caraglia, Chiara Riganti

**Affiliations:** ^1^ Department of Oncology, University of Turin, Turin, Italy; ^2^ Department of Biochemistry, Biophysics and General Pathology, Second University of Naples, Naples, Italy; ^3^ Department of Pharmacy, Federico II University of Naples, Naples, Italy; ^4^ Center for Pharmaceutical Biotechnology and Nanomedicine, Northeastern University, Boston, MA, USA; ^5^ Universidad del Rosario, Facultad de Ciencias Naturales y Matemáticas, RG in Biochemistry and Biotechnology (BIO-BIO), Bogotá, Colombia; ^6^ Sbarro Institute for Cancer Research and Molecular Medicine, Center for Biotechnology, College of Science and Technology, Temple University, Philadelphia, PA, USA; ^7^ Department of Experimental Medicine, Second University of Naples, Naples, Italy

**Keywords:** self-assembling nanoparticles, zoledronic acid, doxorubicin resistance, immunoresistance, immunosuppression

## Abstract

The resistance to chemotherapy and the tumor escape from host immunosurveillance are the main causes of the failure of anthracycline-based regimens in breast cancer, where an effective chemo-immunosensitizing strategy is lacking.

The clinically used aminobisphosphonate zoledronic acid (ZA) reverses chemoresistance and immunoresistance *in vitro*. Previously we developed a nanoparticle-based zoledronic acid-containing formulation (NZ) that allowed a higher intratumor delivery of the drug compared with free ZA *in vivo*. We tested its efficacy in combination with doxorubicin in breast tumors refractory to chemotherapy and immune system recognition as a new combinatorial approach to produce chemo- and immunosensitization.

NZ reduced the IC_50_ of doxorubicin in human and murine chemoresistant breast cancer cells and restored the doxorubicin efficacy against chemo-immunoresistant tumors implanted in immunocompetent mice. By reducing the metabolic flux through the mevalonate pathway, NZ lowered the activity of Ras/ERK1/2/HIF-1α axis and the expression of P-glycoprotein, decreased the glycolysis and the mitochondrial respiratory chain, induced a cytochrome c/caspase 9/caspase 3-dependent apoptosis, thus restoring the direct cytotoxic effects of doxorubicin on tumor cell. Moreover, NZ restored the doxorubicin-induced immunogenic cell death and reversed the tumor-induced immunosuppression due to the production of kynurenine, by inhibiting the STAT3/indoleamine 2,3 dioxygenase axis. These events increased the number of dendritic cells and decreased the number of immunosuppressive T-regulatory cells infiltrating the tumors.

Our work proposes the use of nanoparticle encapsulating zoledronic acid as an effective tool overcoming at the same time chemoresistance and immunoresistance in breast tumors, thanks to the effects exerted on tumor cell and tumor-infiltrating immune cells.

## INTRODUCTION

Anthracycline-based regimens are widely used as neo-adjuvant and adjuvant chemotherapy against breast cancers [1, 2]. The main drawbacks of anthracyclines like doxorubicin are the onset of cardiotoxicity [3, 4] and the onset of drug resistance that makes chemotherapy progressively ineffective. Doxorubicin, as well as the other anthracyclines, are substrates of ATP binding cassette (ABC) transporters, such as P-glycoprotein (Pgp) and multidrug resistance related protein 1 (MRP1), which efflux the drugs outside the tumor cell, thus lowering their cytotoxicity [5].

Liposomal doxorubicin has been successfully employed to circumvent cardiotoxicity [6], but it is not effective against doxorubicin-resistant breast tumors [7]. The co-administration of chemosensitizing agents, such as ABC transporter inhibitors of natural or synthetic origin, has obtained promising results *in vitro* [8–11]. This approach, however, failed to overcome drug resistance *in vivo*, for the low specificity and high toxicity of the chemosensitizing agents [10, 12].

A second criticism of doxorubicin-resistant tumors is their simultaneous resistance to chemotherapy and immune system. In drug-sensitive tumors, doxorubicin induces an immunogenic cell death, by promoting the plasma membrane exposure of the protein calreticulin (CRT), which activates the local dendritic cells (DCs) to phagocytize tumor cells and stimulates the subsequent expansion of anti-tumor CD8^+^ T-lymphocytes [13]. These mechanisms work in doxorubicin-sensitive tumors, not in doxorubicin-resistant ones: here Pgp hinders the doxorubicin-mediated immunogenic cell death by rapidly effluxing the drug and by inhibiting the immunosensitizing function of CRT [14, 15]. Besides escaping the DC-mediated immunosurveillance, drug-resistant tumors show also highly basal synthesis of kynurenine [16, 17], an immunosuppressive catabolite of tryptophan that is produced by indoleamine 2,3-dioxygenase (IDO) [18]. Kynurenine reduces the proliferation and survival of CD4^+^and CD8^+^ T-lymphocytes [17], and promotes the expansion of the immunosuppressive T-regulatory (Treg) cells [16], favoring the immunoescape of drug-resistant tumors.

Since an active immune system plays an important role in limiting the growth and relapse of breast tumors [19–21], chemo-immunotherapy-based approaches are under intensive investigation for these cancers [22–24]. An “ideal” doxorubicin-based regimen for breast cancer should circumvent the tumor drug resistance and prevent at the same time the tumor-induced immunoresistance and immunosuppression.

We previously demonstrated that zoledronic acid (ZA), a clinically used aminobisphosphonate that inhibits the farnesyl pyrophosphate synthase (FPPS) step in the mevalonate pathway [25], is an effective chemo-immunosensitizing agent in doxorubicin-resistant cell lines *in vitro* [16, 26]. When administered as free agent, however, ZA is avidly taken by bone and reaches insufficient intra-tumor concentrations [27]. To overcome this criticism we developed self-assembling nanoparticles (NPs) encapsulating ZA (here called NZ): compared to free ZA, NZ exhibited increased intra-tumor delivery of the aminobisphosphonate [28, 29] and increased anti-proliferative effects against tumors implanted in immunodeficient animals [30–33]. Moreover, the self-assembling feature of these NPs makes them suitable for clinical applications, overcoming the issues generally associated with the scale-up and clinical use of NP formulations [28].

In this work, we investigated whether NZ - in combination with doxorubicin - overcomes chemoresistance and immunoresistance of breast tumors implanted in immunocompetent mice, rescuing the anthracycline's efficacy in refractory breast cancers.

## RESULTS

### NZ reduces the resistance to doxorubicin in breast cancer cells and the growth of chemoresistant tumors

**Figure 1:**
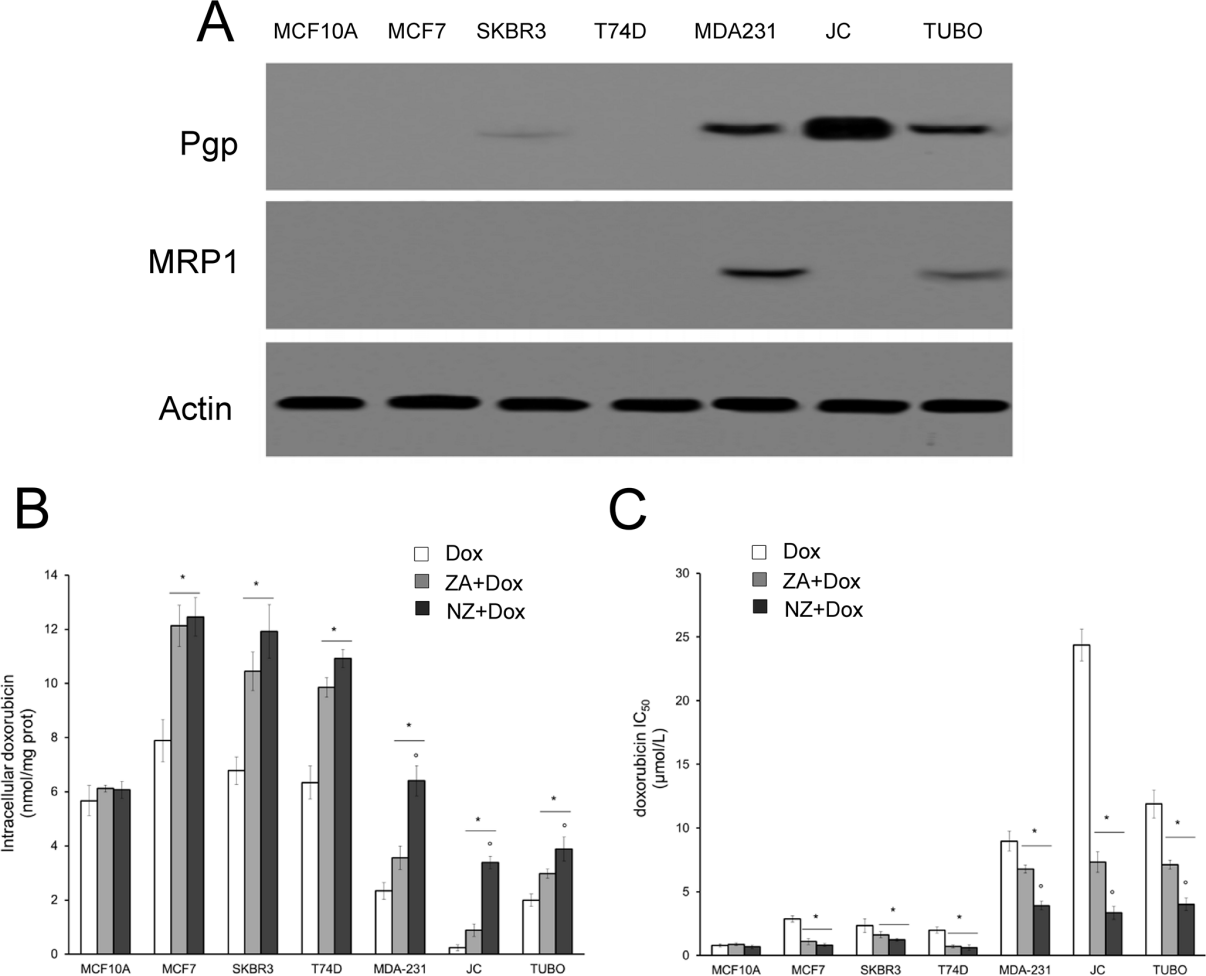
NZ reverses doxorubicin resistance in breast cancer cells Human non transformed breast epithelial MCF10A cells, human breast cancer MCF7, SKBR3, T74D, MDA-MB-231 cells, murine mammary cancer TUBO and JC cells were subjected to the following investigations. (**A**) Western blot analysis of Pgp and MRP1. The actin expression was used as control of equal protein loading. The figure is representative of 3 experiments with similar results. (**B**) Cells were incubated for 24 h with 5 μmol/L doxorubicin (Dox), 1 μmol/L zoledronic acid (ZA) for 24 h followed by 5 μmol/L doxorubicin for additional 24 h (ZA + Dox), 1 μmol/L self-assembling ZA formulation (NZ) for 24 h followed by 5 μmol/L doxorubicin for additional 24 h (NZ + Dox). The intracellular content of doxorubicin was measured spectrofluorimetrically in duplicate (*n* = 4). Data are presented as means ± SD. Versus Dox: *p < 0.001; NZ + Dox versus ZA + Dox: °*p* < 0.01. (**C**) Cells were left untreated or incubated for 72 h in the presence of 1 μmol/L ZA or NZ; different concentrations (1 nmol/L, 10 nmol/L, 100 nmol/L, 1 μmol/L, 10 μmol/L, 100 μmol/L, 1 mmol/L) of doxorubicin (Dox) were added in the last 48 h. Sample were then stained in quadruplicate with the neutral red solution (*n* = 4). IC_50_ was calculated as the concentration of doxorubicin that kills 50% of cells. Data are presented as means ± SD. Versus Dox: *p < 0.05; NZ + Dox versus ZA + Dox: °*p* < 0.005.

We first tested the chemosensitizing effects of NZ and free ZA in a panel of human and murine breast cancer cell lines, showing different expression of the doxorubicin efflux transporters Pgp and MRP1 (Figure 1A). NZ and ZA increased the doxorubicin intracellular retention (Figure 1B) and lowered the doxorubicin IC_50_ (Figure 1C), according to the number of viable cells positive for the neutral red staining after 72 h of treatment: these effects were specific for tumor cells, since they did not occur in the non-transformed MCF10A epithelial cells. NZ was as effective as ZA in the cell lines with low Pgp levels (i.e. MCF7, SKBR3, T74D cells) and significantly more effective than ZA in the cell lines with high Pgp levels (i.e. MDA-MB-231, JC, TUBO cells), suggesting that it was an effective chemosensitizing agent in doxorubicin-resistant breast cancer cells.

**Figure 2:**
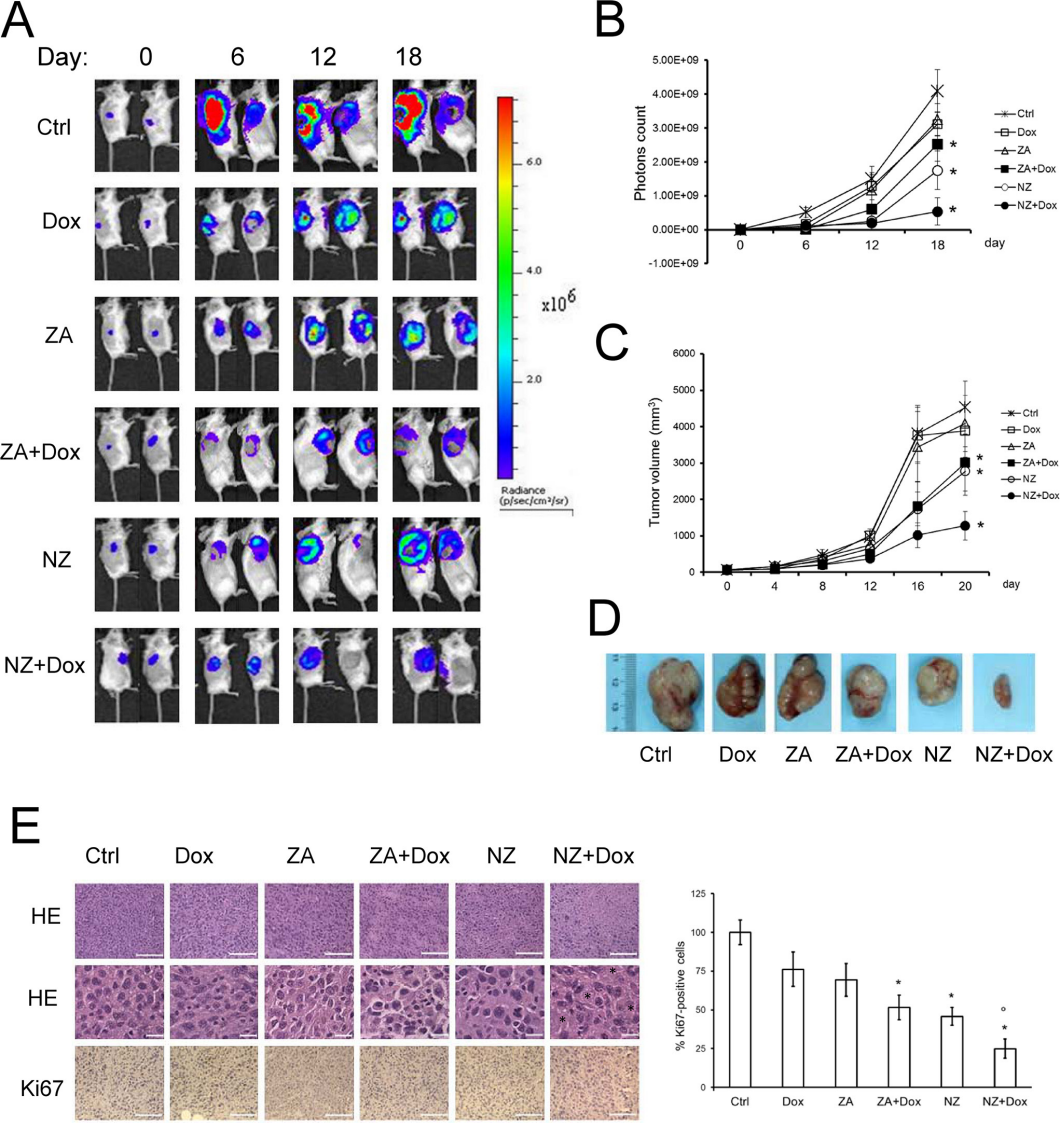
The association of NZ and doxorubicin reduces the growth of chemoresistant tumors Six weeks-old female BALB/c mice bearing 60 mm^3^ JC-luc tumors were randomly divided into the following groups (10 mice/group): 1) Ctrl group, treated with 0.1 mL saline solution i.v. at day 3, 9, 15; 2) Dox group, treated with 5 mg/kg doxorubicin i.v. at day 3, 9, 15; 3) ZA group, treated with 20 μg/mouse ZA i.v. at day 2, 8, 14; 4) ZA + Dox group, treated with 20 μg/mouse ZA i.v. at day 2, 8, 14 followed by 5 mg/kg doxorubicin i.v. at day 3, 9, 15; 5) NZ group, treated with 20 μg/mouse self-assembling ZA formulation i.v. at day 2, 8, 14; 6) NZ + Dox group, treated with 20 μg/mouse NZ i.v. at day 2, 8, 14 followed by 5 mg/kg doxorubicin i.v. at day 3, 9, 15. (**A**) Representative *in vivo* bioluminescence imaging, performed at day 0, 6, 12, 18 after randomization. (**B**) Photons quantification of tumors according to the imaging data. Data are presented as means ± SD. Versus Ctrl group: *p < 0.01. (**C**) Tumor growth was monitored daily by caliper measurement. Data are presented as means ± SD. Versus Ctrl group: *p < 0.005. (**D**) Photograph of representative tumors from each treatment group after mice sacrifice. (**E**) Sections of tumors from each group of animals were stained with hematoxylin and eosin (HE; upper panel: 20× objective; lower panel: 63× objective; stars: necrotic areas) or immunostained for Ki67 (63× objective). Nuclei were counter-stained with hematoxylin. Bar = 10 μm. The photographs are representative of sections from 5 tumors/group. The percentage of proliferating cells was determined by the ratio of Ki67-positive nuclei and the total nuclei (hematoxylin-positive nuclei), by counting sections from 5 animals of each group (108–73 nuclei/field). Ctrl group percentage was considered 100%. Data are presented as means + SD. Versus Ctrl group: *p < 0.05; versus Dox group: °*p* < 0.002.

In the subsequent set of experiments, we focused on the JC model, a constitutively doxorubicin-resistant cell line over-expressing Pgp and syngeneic with BALB/c mice [34]. JC cells stably transduced with a luciferase expression vector (JC-luc clone) were implanted in immunocompetent animals. As shown by the *in vivo* bioluminescence imaging (Figure 2A–2B), by the manual measurement of tumor growth (Figure 2C) and by the tumor gross pathology (Figure 2D and Table 1), doxorubicin and ZA alone did not reduce tumor progression. The combination of ZA and doxorubicin, as well as NZ alone, produced a small reduction of tumor growth (Figure 2A–2D and Table 1) and decreased tumor cell proliferation, as revealed by the Ki67 staining (Figure 2E). The association of NZ and doxorubicin had the strongest effects on the tumor growth (Figure 2A–2D and Table 1); such association reduced tumor cellularity and proliferation, and induced the appearance of intra-tumor necrotic areas (Figure 2E). This combination did not induce more damage on liver, heart and kidney compared to the other treatments, as suggested by the hematochemical parameters of hepatotoxicity (lactate dehydrogenase LDH, aspartate aminotransferase AST, alanine aminotransferase ALT, alkaline phosphatase AP), cardiotoxicity (creatine phosphokinase CPK), nefrotoxicity (creatinine; Table 2). NPs without ZA (blank NPs) did not exert any chemosensitizing effects *in vitro* (Supplementary Figure 1A–1B) and *in vivo* (Supplementary Figure 1C), and were not further evaluated in the study.

**Table 1:** Measurement of animals weight, tumor weight and tumor growth inhibition

	Ctrl	Dox	ZA	ZA + dox	NZ	NZ + dox
**Final mice weight (g)**	20.28±3.46	20.19± 3.81	21.09 ±2.33	20.11 ±1.29	20.01 ±2.74	19.68 ±1.82
**Final tumor weight (g)**	3.51±0.72	3.18 ±0.91	3.02 ±0.39	2.28 ±0.31*	2.25 ±0.42*	1.12 ±0.24*
**% inhibition rate**		9.40%	13.96%	35.04%*	35.90%*	68.09%*

Animals (*n* = 10/group) were treated as reported under Materials and methods. The percentage of inhibition rate was calculated as it follows: (average tumor weight of control group – average tumor weight of test group)/average tumor weight of control group × 100%. Significance versus Ctrl group: *p < 0.01.

**Table 2:** Hematochemical parameters of animals

	Ctrl	Dox	ZA	ZA + Dox	NZ	NZ + Dox
**LDH (U/L)**	6052±1761	7098±1872	6677±1512	7551±1927	7091±1872	7381±1724
**AST (U/L)**	235± 81	289± 77	322± 81	331± 67	284± 62	312± 86
**ALT (U/L)**	27.5± 4.5	29.8± 6.3	28.6± 1.9	30.6± 3.4	28.7± 3.4	28.6± 3.4
**AP (U/L)**	81 ±16	74 ±11	79 ±11	81 ±18	82 ±13	79 ±13
**CPK (U/L)**	467±109	670±156	681±208	608±192	679±129	689±132
**Creatinine (mg/L)**	0.034+0.004	0.036±0.005	0.038±0.004	0.040±0.005	0.039±0.004	0.037±0.007

Animals (*n* = 10/group) were treated as reported under Materials and methods. Blood was collected immediately after mice euthanasia and analyzed for lactate dehydrogenase (LDH), aspartate aminotransferase (AST), alanine aminotransferase (ALT), alkaline phosphatase (AP), creatine phosphokinase (CPK), creatinine. There were not statistically significant differences among each group of treatment.

### NZ lowers the Pgp levels in chemoresistant tumors

**Figure 3:**
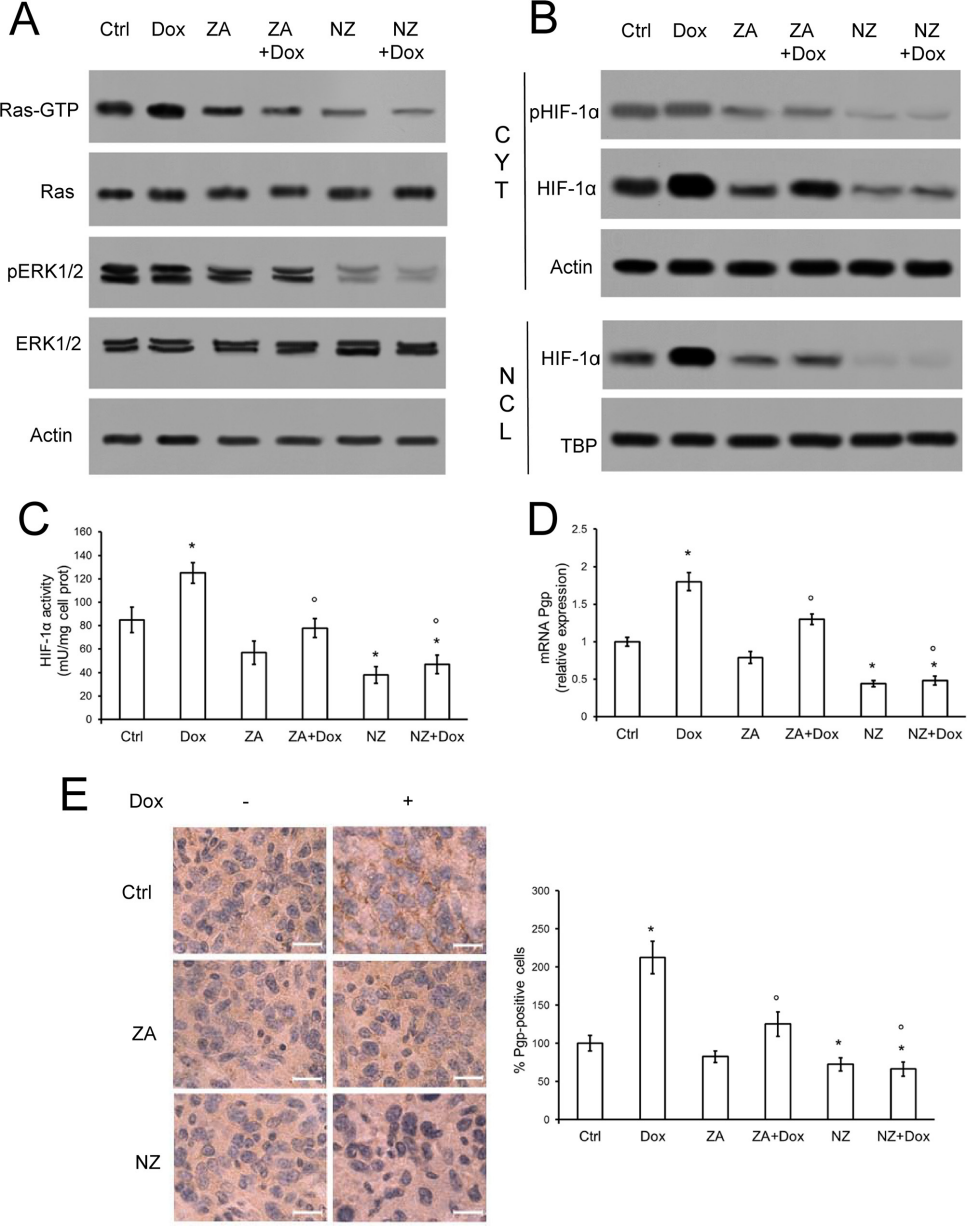
NZ decreases the activation of Ras/ERK1/2/HIF1α axis and the expression of Pgp in chemoresistant tumors Six weeks-old female BALB/c mice bearing 60 mm^3^ JC-luc tumors were randomly divided into the following groups (10 mice/group): 1) Ctrl group, treated with 0.1 mL saline solution i.v. at day 3, 9, 15; 2) Dox group, treated with 5 mg/kg doxorubicin i.v. at day 3, 9, 15; 3) ZA group, treated with 20 μg/mouse ZA i.v. at day 2, 8, 14; 4) ZA + Dox group, treated with 20 μg/mouse ZA i.v. at day 2, 8, 14 followed by 5 mg/kg doxorubicin i.v. at day 3, 9, 15; 5) NZ group, treated with 20 μg/mouse self-assembling ZA formulation i.v. at day 2, 8, 14; 6) NZ + Dox group, treated with 20 μg/mouse NZ i.v. at day 2, 8, 14 followed by 5 mg/kg doxorubicin i.v. at day 3, 9, 15. (**A**) Tumor extracts were subjected to the Western blot analysis for Ras-GTP, total Ras, phospho(Thr202/Tyr204, Thr185/Tyr187)-ERK1/2, total ERK1/2. The actin expression was used as control of equal protein loading. The figure is representative of 4 tumors per each group. (**B**) Cytosolic (CYT) and nuclear (NCL) fractions were separated from tumor extracts and analyzed for the expression of phospho (Ser) HIF-1α and HIF-1α by Western blotting. The actin and TBP expressions were used as controls of equal protein loading in each fraction. The figure is representative of 4 tumors per each group. (**C**) HIF-1α activity was measured in nuclear extracts from each tumor (*n* = 10/group) by ELISA. Data are presented as means + SD. Versus Ctrl group: *p < 0.05; versus Dox group: °*p* < 0.05. (**D**) *Pgp* mRNA levels were detected in each tumor extract (*n* = 10/group) by qRT-PCR. Data are presented as means + SD. Versus Ctrl group: *p < 0.05; versus Dox group: °*p* < 0.01. (**E**) Sections of tumors from each group of animals were immunostained for Pgp. Nuclei were counter-stained with hematoxylin. Bar = 10 μm (63× objective). The photographs are representative of sections from 5 tumors/group. The percentage of Pgp-positive cells was determined by analyzing sections from 5 animals of each group (110–72 cells/field), using Photoshop program. The intensity of Ctrl group was considered 100%. Data are presented as means + SD. Versus Ctrl group: *p < 0.05; versus Dox group: °*p* < 0.001.

Since the chemosensitizing effects were more related to the presence of Pgp than MRP1, which was present in only two of Pgp-containing cell lines and was less expressed than Pgp, we focused our attention on the modulation of the latter. In line with recent findings on lung cancer [33], NZ and - at a lesser extent ZA - lowered the activity of Ras and Ras-downstream effectors ERK1/2 (Figure 3A), the amount, phosphorylation, nuclear translocation (Figure 3B) and activity (Figure 3C) of HIF-1α, the transcription of the HIF-1α-target gene *Pgp* (Figure 3D) and the amount of Pgp protein (Figure 3E) in JC tumor extracts. The effects of NZ on Ras/ERK1/2/HIF-1α/Pgp axis was likely due to the strong reduction in the synthesis of FPP (Supplementary Figure 2), a critical metabolite for Ras activity [35]. ZA caused a significant but weaker decrease in FPP levels (Supplementary Figure 2); doxorubicin did not alter FPP synthesis (Supplementary Figure 2) or Ras/ERK1/2 activity in untreated, ZA-treated and NZ-treated tumors (Figure 3A). The anthracycline increased HIF-1α amount and nuclear translocation, (Figure 3B–3C) and Pgp levels (Figure 3D) in tumor extracts: these effects were fully prevented by NZ, which decreased HIF-1α and Pgp amount below the control (Figure 3B–3E).

### NZ impairs the energy metabolism and the mitochondrial functions, inducing cytochrome c/caspase 9/caspase 3-dependent apoptosis in chemoresistant tumors

**Figure 4:**
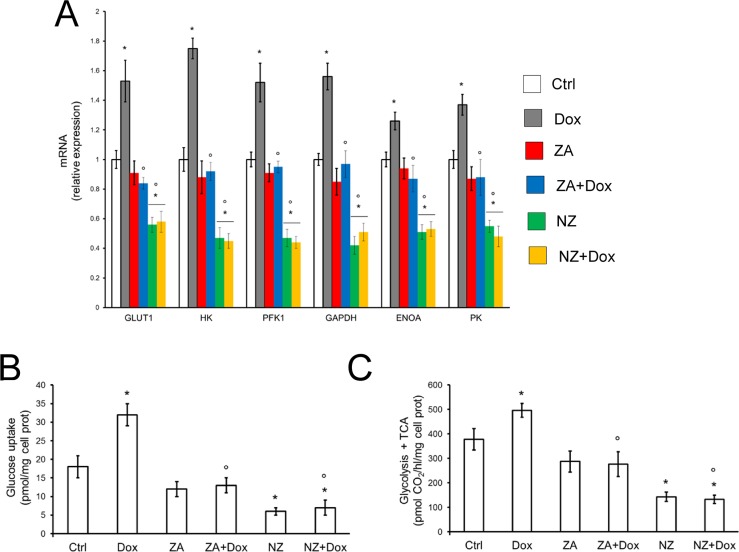
NZ reduces the glucose metabolism in chemoresistant cells (**A**) Six weeks-old female BALB/c mice bearing 60 mm^3^ JC-luc tumors were randomly divided into the following groups (10 mice/group): 1) Ctrl group, treated with 0.1 mL saline solution i.v. at day 3, 9, 15; 2) Dox group, treated with 5 mg/kg doxorubicin i.v. at day 3, 9, 15; 3) ZA group, treated with 20 μg/mouse ZA i.v. at day 2, 8, 14; 4) ZA + Dox group, treated with 20 μg/mouse ZA i.v. at day 2, 8, 14 followed by 5 mg/kg doxorubicin i.v. at day 3, 9, 15; 5) NZ group, treated with 20 μg/mouse self-assembling ZA formulation i.v. at day 2, 8, 14; 6) NZ + Dox group, treated with 20 μg/mouse NZ i.v. at day 2, 8, 14 followed by 5 mg/kg doxorubicin i.v. at day 3, 9, 15. Tumor extracts (*n* = 10/group) were analyzed for the mRNA levels of *GLUT1, HK, PFK1, GAPDH, ENOA, PK* by qRT-PCR. Data are presented as means + SD. Versus Ctrl group: *p < 0.001; versus Dox group: °*p* < 0.001. (**B**) JC cells were grown in fresh medium (Ctrl) or medium containing 5 μmol/L doxorubicin (Dox, 24 h), 1 μmol/L zoledronic acid (ZA, 48 h), 1 μmol/L ZA for 24 h followed by 5 μmol/L doxorubicin for additional 24 h (ZA + Dox), 1 μmol/L self-assembling ZA formulation (NZ, 48 h), 1 μmol/L NZ for 24 h followed by 5 μmol/L doxorubicin for additional 24 h (NZ + Dox). The uptake of 2-deoxy-D-[^3^H]-glucose was measured by cell radiolabeling and quantified by liquid scintillation. Data are presented as means + SD (*n* = 3). Versus Ctrl: *p < 0.001; versus Dox: °*p* < 0.002. (**C**) JC cells were incubated as reported in (**B**). The glucose flux through glycolysis and TCA cycle was measured in cells radiolabeled with [6–^14^C]-glucose. Data are presented as means + SD (*n* = 3). Versus Ctrl: *p < 0.001; versus Dox: °*p* < 0.001.

Doxorubicin increased the mRNA levels of the HIF-1α-target genes involved in the glucose uptake and glycolytic flux, such as glucose transporter 1 (*GLUT1*), hexokinase (*HK*), phosphofructokinase-1 (*PFK1*), glyceraldehyde 3-phosphate dehydrogenase (*GAPDH*), enolase A (*ENOA*), pyruvate kinase (*PK*) in tumor extracts (Figure 4A). By contrast, NZ, either alone or in the presence of doxorubicin, significantly reduced the levels of these mRNAs in JC tumors (Figure 4A). In keeping with these effects, doxorubicin increased and NZ decreased the glucose uptake (Figure 4B) and the glucose flux through glycolysis and tricarboxylic acid (TCA) cycle (Figure 4C) in JC cells.

**Figure 5:**
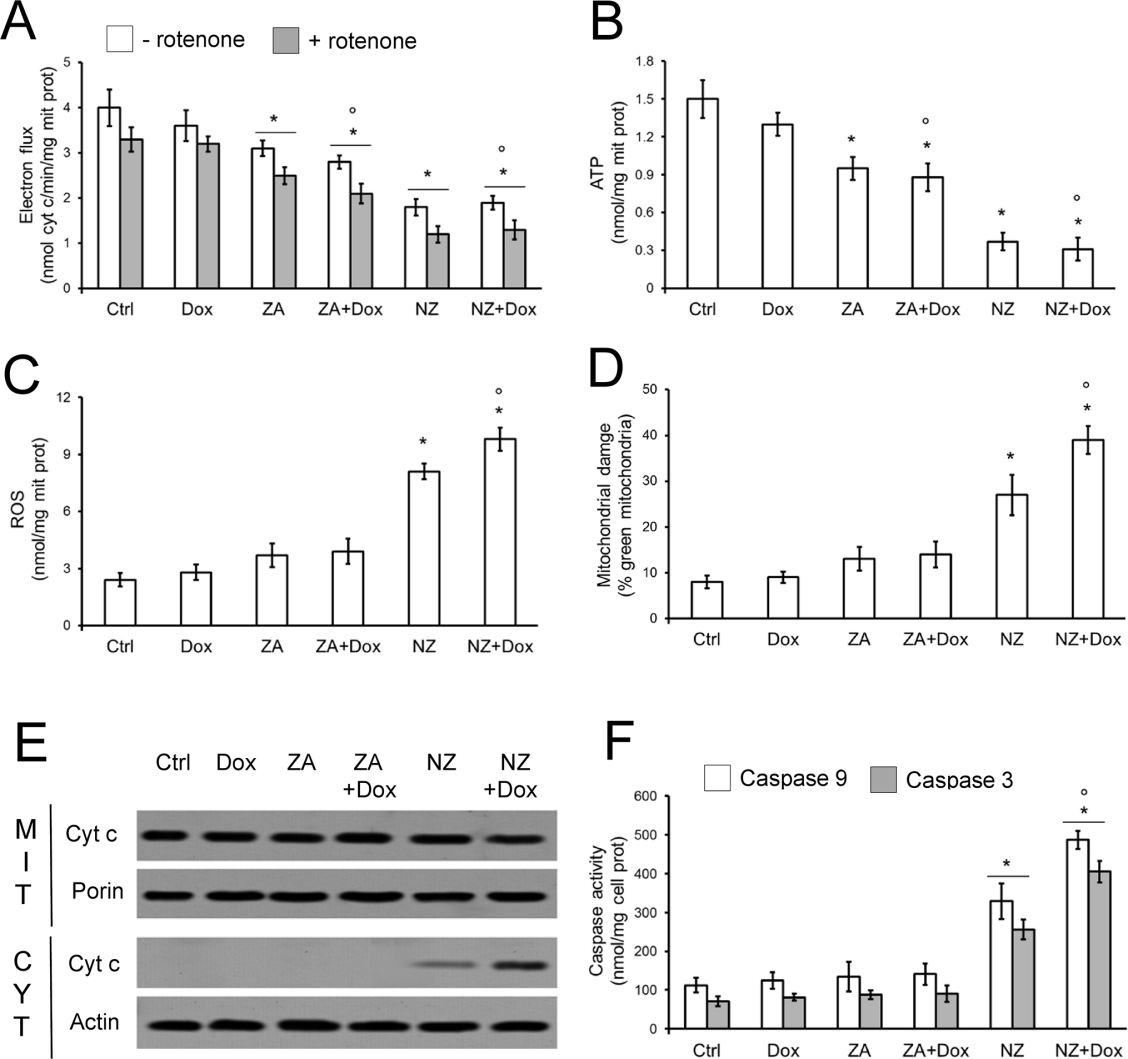
NZ impairs the mitochondrial metabolism and induces apoptotic death in chemoresistant cells JC cells were grown in fresh medium (Ctrl) or medium containing 5 μmol/L doxorubicin (Dox, 24 h), 1 μmol/L zoledronic acid (ZA, 48 h), 1 μmol/L ZA for 24 h followed by 5 μmol/L doxorubicin for additional 24 h (ZA + Dox), 1 μmol/L self-assembling ZA formulation (NZ, 48 h), 1 μmol/L NZ for 24 h followed by 5 μmol/L doxorubicin for additional 24 h (NZ + Dox). (**A**) The electron flux between Complex I and III, either ubiquinone-independent (i.e. in the absence of the Complex I inhibitor rotenone, 50 μmol/L) or ubiquinone-dependent (i.e. in the presence of rotenone), was measured spectrophotometrically in isolated mitochondria. Data are presented as means + SD (*n* = 3). Versus Ctrl: *p < 0.05; versus Dox: °*p* < 0.01. (**B**) ATP levels in isolated mitochondria were measured by a chemiluminescence-based assay. Data are presented as means + SD (*n* = 3). Versus Ctrl: *p < 0.01; versus Dox: °*p* < 0.01. (**C**) Intramitochondrial ROS levels were measured spectrofluorimetrically in triplicate using the DCFDA-AM probe. Data are presented as means + SD (*n* = 3). Versus Ctrl: *p < 0.05; versus Dox: °*p* < 0.001. (**D**) The mitochondrial membrane potential was assessed by the JC-1 staining method. The percentage of green versus red mitochondria was considered an index of mitochondrial depolarization and permeability transition. Data are presented as means + SD (*n* = 3). Versus Ctrl: *p < 0.05; versus Dox: °*p* < 0.001. (**E**) Mitochondrial (MIT) and cytosolic (CYT) extracts were analyzed for the levels of cytochrome c by Western blotting. The porin and actin expressions were used as controls of equal protein loading in each fraction. The figure is representative of 3 experiments. (**F**) The activities of caspase 9 and 3 were measured spectrofluorimetrically in the cell lysates. Data are presented as means + SD (*n* = 3). Versus Ctrl: *p < 0.005; versus Dox: °*p* < 0.005.

Whereas doxorubicin had no effect on mitochondrial electron flux (Figure 5A) and ATP production (Figure 5B), NZ decreased these parameters (Figure 5A–5B). NZ effect was likely due to the decreased amount of the mitochondrial electron transporter ubiquinone (Supplementary Figure 3), whose synthesis depends on the mevalonate pathway, since it contains an isoprenoid tail essential for its activity [36]. The impairment of the mitochondrial energy metabolism induced by NZ was paralleled by increased reactive oxygen species (ROS; Figure 5C), mitochondrial depolarization (Figure 5D), release of cytochrome c in the cytosol (Figure 5E), activation of caspase 9 and caspase 3 (Figure 5F).

ZA, which decreased the energy metabolism (Figure 4, Figure 5A–5B) and ubiquinone synthesis (Supplementary Figure 3) less than NZ, poorly increased ROS levels and mitochondrial depolarization, without eliciting cytochrome c release and caspases activation (Figure 5C–5F). Similarly, doxorubicin was devoid of any mitochondrial-damaging or pro-apoptotic effects in JC cells. Only the pre-treatment with NZ induced all these events in doxorubicin-treated cells (Figure 5C–5F). The activation of caspase 9 and caspase 3 by NZ was time-dependent (Supplementary Figure 4A–4D); the addition of doxorubicin further increased caspases activities in cells incubated with NZ for at least 12 h (Supplementary Figure 4A–4D; Figure 5F).

### The association of NZ and doxorubicin restores the recognition of resistant tumors by the host immune system

**Figure 6:**
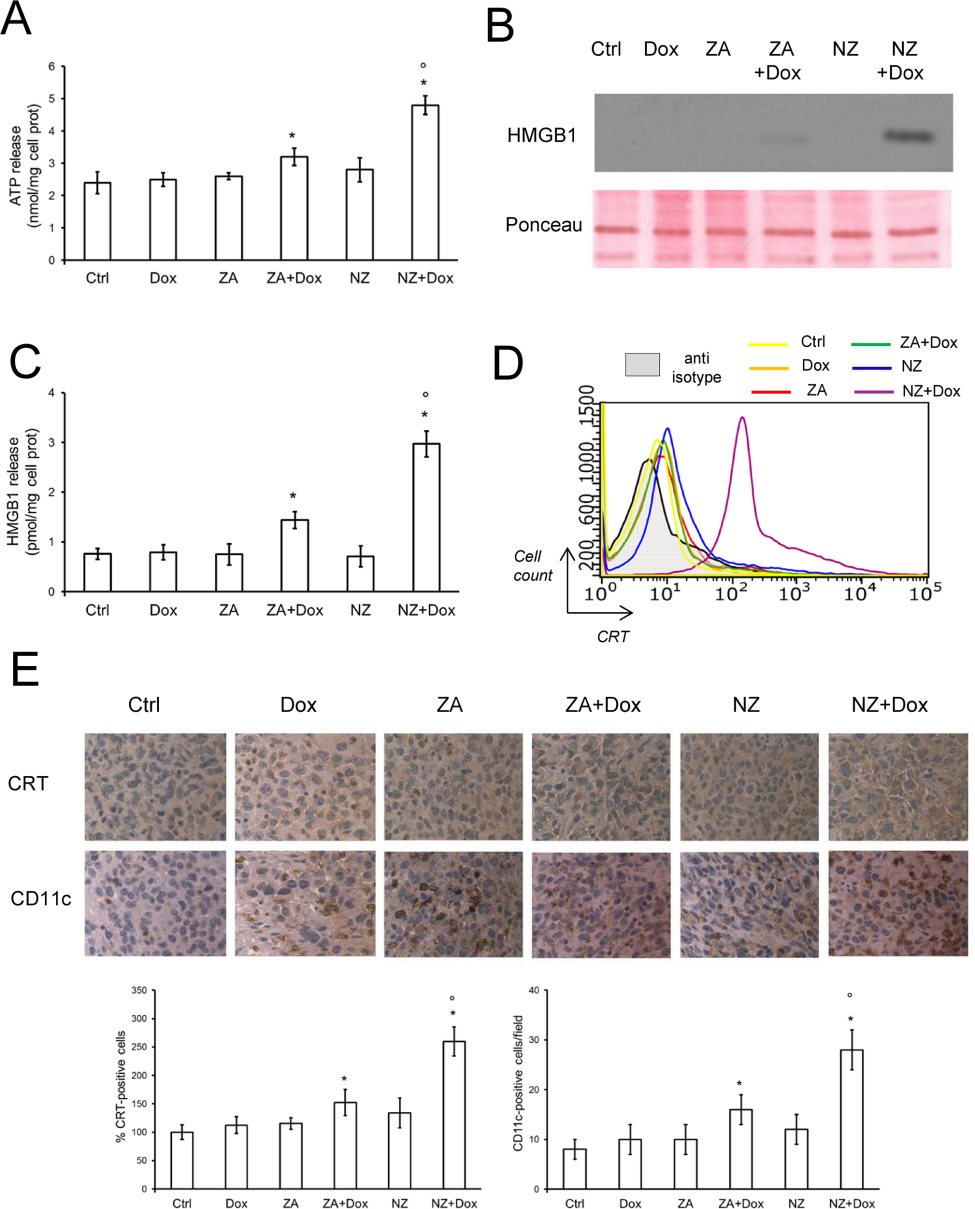
NZ restores the doxorubicin-induced immunogenic death in chemoresistant tumors (**A**) JC cells were grown in fresh medium (Ctrl) or medium containing 5 μmol/L doxorubicin (Dox, 24 h), 1 μmol/L zoledronic acid (ZA, 48 h), 1 μmol/L ZA for 24 h followed by 5 μmol/L doxorubicin for additional 24 h (ZA + Dox), 1 μmol/L self-assembling ZA formulation (NZ, 48 h), 1 μmol/L NZ for 24 h followed by 5 μmol/L doxorubicin for additional 24 h (NZ + Dox). The extracellular release of ATP was measured by a chemiluminescence-based assay. Data are presented as means + SD (*n* = 3). Versus Ctrl: *p < 0.05; versus Dox: °*p* < 0.001. (**B**) Western blot analysis of HMGB1 in the cell supernatants. Red Ponceau staining was used to check the equal loading of proteins. The figure is representative of 3 experiments. (**C**) The extracellular release of HMGB1 was measured by ELISA. Data are presented as means + SD (*n* = 3). Versus Ctrl: *p < 0.005; versus Dox: °*p* < 0.001. (**D**) Surface calreticulin (CRT) was measured by flow cytometry in duplicate. The figure is representative of 3 experiments. Anti-isotype: incubation with non immune isotypic antibody, included as negative control. (**E**) Six weeks-old female BALB/c mice bearing 60 mm^3^ JC-luc tumors were randomly divided into the following groups (10 mice/group): 1) Ctrl group, treated with 0.1 mL saline solution i.v. at day 3, 9, 15; 2) Dox group, treated with 5 mg/kg doxorubicin i.v. at day 3, 9, 15; 3) ZA group, treated with 20 μg/mouse ZA i.v. at day 2, 8, 14; 4) ZA + Dox group, treated with 20 μg/mouse ZA i.v. at day 2, 8, 14 followed by 5 mg/kg doxorubicin i.v. at day 3, 9, 15; 5) NZ group, treated with 20 μg/mouse self-assembling ZA formulation i.v. at day 2, 8, 14; 6) NZ + Dox group, treated with 20 μg/mouse NZ i.v. at day 2, 8, 14 followed by 5 mg/kg doxorubicin i.v. at day 3, 9, 15. Sections of tumors from each group of animals were immunostained for CRT or CD11c, a marker of dendritic cells. Nuclei were counter-stained with hematoxylin. Bar = 10 μm (63× objective). The photographs are representative of sections from 5 (for CRT) or 10 (for CD11c) tumors/group. The percentage of CRT-positive cells was determined by analyzing sections from 5 animals of each group (108–76 cells/field), using Photoshop program. The intensity of Ctrl group was considered 100%. The number of CD11c-positive cells/field was calculated by analyzing sections from 10 animals of each group (114–71 cells/field), using ImageJ software (http://imagej.nih.gov/ij/). Data are presented as means + SD. Versus Ctrl group: *p < 0.05; versus Dox group: °*p* < 0.001.

We previously demonstrated that the combination of ZA and doxorubicin restored the immunogenic effects of the anthracycline in mildly chemoresistant cells *in vitro*, where doxorubicin alone was ineffective [26]. However, in the highly chemoresistant JC cells, the association of doxorubicin and ZA elicited low pro-immunogenic effects, as revealed by the very weak extracellular release of ATP (Figure 6A) and high mobility group binding protein 1 (HMGB1; Figure 6B–6C), and by the absence of CRT exposure on JC cell surface (Figure 6D), considered as indexes of immunogenic death [37]. ZA and NZ alone did not elicit these effects as well. Interestingly, the association of NZ and doxorubicin was the only treatment that significantly increased the extracellular release of ATP and HMGB1 (Figure 6A–6C), and the exposure of CRT on JC cell surface (Figure 6D). NZ increased the release of ATP and HMGB1 if incubated at least 48 h before doxorubicin; shorter incubations did not elicited these effects (Supplementary Figure 5A–5H).

In keeping with these findings, the number of cells positive for CRT and the number of DCs present within JC tumors, revealed by the staining for the DC-marker CD11c, were markedly increased in animals exposed to NZ and doxorubicin (Figure 6E).

### NZ lowers the tumor-induced kynurenine production by reducing the activation of STAT3 and decreases the number of intra-tumor Treg lymphocytes

**Figure 7:**
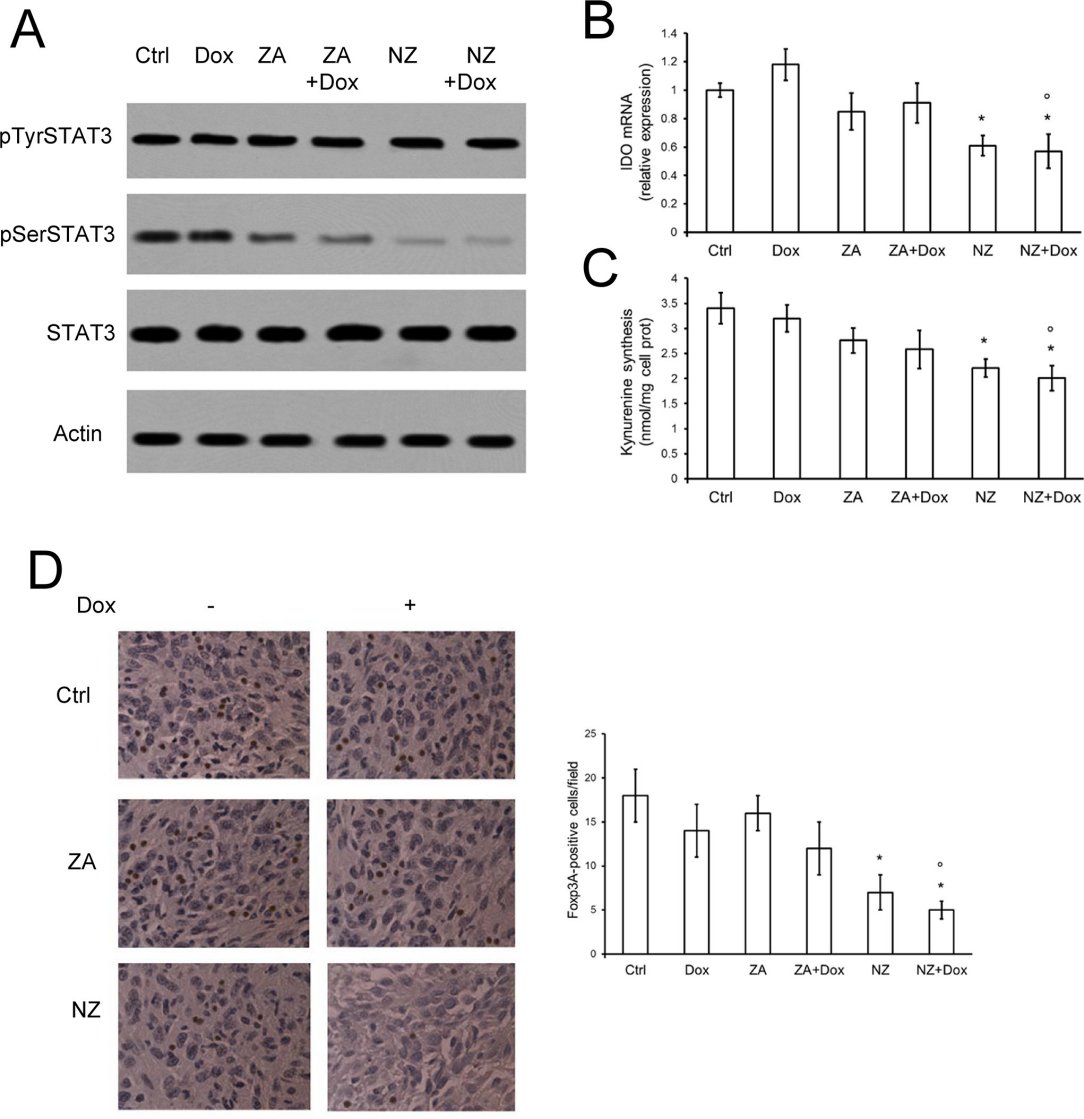
NZ counteracts the tumor-induced immunosuppressive microenvironment Six weeks-old female BALB/c mice bearing 60 mm^3^ JC-luc tumors were randomly divided into the following groups (10 mice/group): 1) Ctrl group, treated with 0.1 mL saline solution i.v. at day 3, 9, 15; 2) Dox group, treated with 5 mg/kg doxorubicin i.v. at day 3, 9, 15; 3) ZA group, treated with 20 μg/mouse ZA i.v. at day 2, 8, 14; 4) ZA + Dox group, treated with 20 μg/mouse ZA i.v. at day 2, 8, 14 followed by 5 mg/kg doxorubicin i.v. at day 3, 9, 15; 5) NZ group, treated with 20 μg/mouse self-assembling ZA formulation i.v. at day 2, 8, 14; 6) NZ + Dox group, treated with 20 μg/mouse NZ i.v. at day 2, 8, 14 followed by 5 mg/kg doxorubicin i.v. at day 3, 9, 15. (**A**) Tumor extracts were subjected to the Western blot analysis for phospho(Tyr705)-STAT3, phospho(Ser727)-STAT3, total STAT3. The actin expression was used as control of equal protein loading. The figure is representative of 4 tumors per each group. (**B**) *IDO* mRNA levels were detected in each tumor extract (*n* = 10/group) by qRT-PCR. Data are presented as means + SD. Versus Ctrl group: *p < 0.01; versus Dox group: °*p* < 0.01. (**C**) The release of kynurenine in the tumor supernatants was measured spectrophotometrically (*n* = 10/group). Data are presented as means + SD. Versus untreated Ctrl group: *p < 0.02; versus Dox group: °*p* < 0.02. (**D**) Sections of tumors from each group of animals were immunostained for Foxp3A, a marker of Treg cells. Nuclei were counter-stained with hematoxylin. Bar = 10 μm (63× objective). The photographs are representative of sections from 10 tumors/group. The number of Foxp3A-positive cells/field was calculated by analyzing sections from 10 animals of each group (111–75 cells/field), using ImageJ software (http://imagej.nih.gov/ij/). Data are presented as means + SD. Versus Ctrl group: *p < 0.02; versus Dox group: °*p* < 0.005.

As observed previously, ZA can lower *in vitro* the synthesis of the immunosuppressive metabolite kynurenine, by inhibiting the Ras/ERK1/2 pathway and the phosphorylation of STAT3 on serine 727 [16]. In JC tumors extracted from mice treated with ZA, however, we did not observe any reduction of phosphorylated STAT3 (Figure 7A), *IDO* mRNA (Figure 7B) and kynurenine production (Figure 7C). These three events were instead detected in tumors derived from NZ-treated animals and were not affected by doxorubicin (Figure 7A–7C). Neither ZA nor NZ, alone and in combination with doxorubicin, modified the amount of intra-tumor CD19^+^, CD4^+^ or CD8^+^ cells (Supplementary Figure 6), suggesting that they did not affect the amount of B- and T-lymphocytes infiltrating the tumor bulk.

High levels of kynurenine have been correlated with the expansion of immunosuppressive Treg cells [16], which favor an immunotolerant and permissive environment for tumor growth [38]. Of note, JC tumors were basally infiltrated of immunosuppressive Treg cells, as documented by the presence of Foxp3A^+^ cells (Figure 7D). ZA and doxorubicin did not modify such infiltrate, which was instead significantly reduced by NZ (Figure 7D). An active immune system was essential to mediate the anti-tumor effect of NZ and to fully rescue doxorubicin's activity: indeed NZ, alone or in combination with doxorubicin, reduced JC tumor growth implanted also in NOD SCID mice (Supplementary Figure 7), but the anti-tumor activity of the combination “NZ plus doxorubicin” was significantly stronger in immunocompetent (Figure 2C) than in immunodeficient (Supplementary Figure 7) animals.

## DISCUSSION

In this work we show that NZ restores the sensitivity to doxorubicin in resistant breast tumors, re-inducing both the “direct” cytotoxic effects – i.e. reducing cell viability and inducing a necro-apoptotic cell death – and the “indirect” effects – i.e. restoring the doxorubicin-induced immunogenic death – exerted by anthracyclines in responsive tumors.

NZ produced a significant chemosensitization in breast cancer cell lines that differ for the expression of estrogen receptor, progesterone receptor and human epidermal growth factor receptor 2 (Her2) [39]. Worthy of note, NZ exerted strong chemosensitizing effects in triple negative breast cancer cells such as MDA-MB-231 cells. This result is important in a translational perspective since the first-line therapy in triple negative breast cancers is chemotherapy, which achieves poor results for the intrinsic chemoresistance of these tumors [40]. Moreover, the chemosensitizing effects of NZ were not species-specific, since they were detected in both human and murine resistant cells.

To validate the efficacy *in vivo* and investigate the molecular mechanisms of the NZ-induced chemosensitization, we focused on the JC model, which was the most chemoresistant cell line *in vitro* and was syngeneic with BALB/c mice [34]. This feature allowed us to set up a model of chemoresistant tumor implanted in immunocompetent animals and to examine the effects of NZ on both tumor and host immune system.

Interestingly, whereas ZA did not reduce the tumor growth, NZ exerted a detectable anti-proliferative effect even when used alone. This result, which is in keeping with previous findings [30, 32], can be explained by the use of NPs that allows a higher delivery of the aminobisphosphonate within the tumor, compared to free ZA [28–33].

By reducing the Ras/ERK1/2 activity, NZ lowered the ERK1/2-dependent phosphorylation and activation of HIF-1α, which was constitutively translocated into the nucleus in JC cells even under normoxia, as it occurs in many chemoresistant cells [26]. Several HIF-1α target genes support cell proliferation and glucose metabolism through the glycolytic pathway [41]. In accord with these experimental evidences, NZ reduced proliferation, down-regulated the expression levels of glycolytic genes and decreased the glucose flux through glycolysis and TCA cycle in our model. In parallel, by reducing the supply of isoprenoid moieties necessary for the electron transporter ubiquinone, it reduced the mitochondrial respiratory chain activity. This global impairment of the energy metabolism determined lower production of ATP and increased ROS levels, which damaged mitochondria and induced a mitochondria-dependent apoptosis. The simultaneous activation of glycolysis and oxidative phosphorylation is a typical feature of chemoresistant cells [42], which require huge amounts of ATP to support the ABC transporters activity. On the other hand, chemoresistant cells are more susceptible to the ATP depletion and to the ROS-induced damage than chemosensitive cells [43]. NZ, by decreasing ATP supply and increasing ROS levels, hits two “Achille's heels” of chemoresistant cells. This mechanism can explain the anti-proliferative effect of NZ alone against chemoresistant breast tumors.

More importantly, NZ restored the anti-tumor efficacy of doxorubicin in JC tumors, which were fully refractory to the drug. In line with the *in vitro* data, the combination of NZ and doxorubicin was more effective than the combination of ZA and doxorubicin. Since Pgp is a target gene of HIF-1α [41], the circumvention of doxorubicin resistance in NZ-treated animals was likely due to the down-regulation of the HIF-1α-mediated transcription of *Pgp*. It has been reported that doxorubicin activates HIF-1α by decreasing the intracellular availability of iron [44]: this finding is in accord with our scenario, where doxorubicin increased the transcriptional activity of HIF-1α in a Ras/ERK1/2-independent way. Although the intra-tumor concentration of doxorubicin in JC tumors is very low [45], such concentration was sufficient to increase HIF-1α and up-regulate Pgp levels, glycolytic genes levels, glucose uptake and metabolism through glycolysis and TCA cycle. This metabolic signature, which provides JC tumors with ATP and can maintain the ATP-dependent activity of Pgp, was abrogated by NZ.

The low concentration of doxorubicin within JC tumors, however, was not sufficient to impair the mitochondrial respiratory chain and increase the ROS production from Complex I, two events that contribute to the doxorubicin cytotoxicity in drug-sensitive cells [46, 47], but not in drug-resistant ones [45]. NZ, which increased the intracellular retention of doxorubicin in JC cells, restored also these “mitochondrial” effects of the drug. Moreover, NZ alone impaired the mitochondrial energy metabolism. We might speculate that in tumors exposed to NZ and doxorubicin the effects of NZ on mitochondria functions are additive to the effects of doxorubicin: the two drugs strongly reduced the electron flux and ATP synthesis, increased ROS levels and mitochondrial depolarization, activated the cytochrome c/caspase 9/caspase 3-dependent pro-apoptotic axis.

The effects of ZA on HIF-1α transcriptional activity, glucose metabolism and mitochondrial functions were lower than those elicited by NZ. As noted earlier, the lower intra-tumor uptake of free ZA compared to NZ [28–32] limits the ability of the former to reduce the synthesis of FPP and ubiquinone, to inhibit the Ras/ERK1/2/HIF-1α/Pgp axis, to impair the energy metabolism, to fully restore doxorubicin's efficacy *in vivo*.

In partial contrast with our work, the treatment with ZA administered before doxorubicin has not improved the efficacy of the anthracycline in human MDA-MB-436 cells implanted in nude mice [48]. This model, however, differs from the one used in our work for several reasons. First, the use of free ZA instead of NZ reduces the antitumor effects of the aminobisphosphonate, as demonstrated in other tumor models [30, 32]. Second, ZA and doxorubicin target both tumor cells and tumor-associated immune cells [13, 49, 50]: the use of immunodeficient mice did not allow to investigate the effects of ZA and doxorubicin on the host immune system. Our comparison between the anti-tumor effect of NZ in immunocompetent and immunodeficient mice suggested that, although NZ was effective also in immunodeficient animals, in line with previous observations [30–33], it achieved the maximal efficacy in animals with an active immune system. These results suggest that part of the anti-tumor effects of NZ was mediated by the activation of the host immune system against the tumor.

Indeed, we found that NZ restored the antitumor efficacy of doxorubicin also by rescuing its pro-immunogenic effects, as revealed by the increased extracellular release of ATP and HMGB1, and by the increased amount of CRT on the plasma membrane. The concurrent presence of these three events is widely recognized as indexes of immunogenic cell death [37]. In our model, the release of ATP and HMGB1 elicited by NZ started later than the triggering of apoptosis: these findings led to hypothesize that the apoptotic process induced by NZ primes cells for a more severe damage, leading to the release of ATP and HMGB1. We cannot exclude that these molecules are released passively as a consequence of the necro-apoptotic cell death; however, since they are paralleled by the exposure of CRT on cell surface, they can suggest a simultaneous induction of immunogenic cell death.

The exposure of CRT on the cell surface coupled with the reduction of Pgp levels, two events that occurred in JC tumors treated with NZ and doxorubicin, are necessary to trigger the tumor cell phagocytosis by local DCs [15, 26]. The increased amount of DCs infiltrating JC tumors treated with NZ and doxorubicin suggests an increased recruitment of local DCs. Although the presence of DCs within breast tumors has been associated with either anti-tumor or pro-tumor effects, the presence of DCs activated by the release of HMGB1 has been unequivocally correlated with the recruitment of T-lymphocytes recognizing tumor-associated antigens [21]. An immunotherapy based on the only boosting of DCs, however, is ineffective in breast cancer and requires the integration with chemotherapy and/or therapeutic tools that inhibit immunosuppressive lymphocytes [23].

According to our data, NZ can be an excellent immunoadjuvant agent: indeed, besides recruiting local DCs, it lowered the production of the immunosuppressive metabolite kynurenine by the resistant tumors. The expression of IDO, the main enzyme producing kynurenine in resistant cancer cells [17], is controlled by the transcriptional factor STAT3, which is active when phosphorylated on tyrosine 705 and serine 727 [51, 52]. In resistant malignant pleural mesothelioma cells, the Ras/ERK1/2 axis promotes the phosphorylation of STAT3 on serine 727 and increases the transcription of *IDO*; this event is prevented by ZA and reduces the *ex vivo* expansion of immunosuppressive Treg cells [16]. A similar mechanism occurred in JC tumors, where ZA and in particular NZ reduced the phosphorylation of STAT3 on serine 727. The slight reduction of phospho(Ser727) STAT3 elicited by ZA, however, was not sufficient to produce any change in kynurenine synthesis. By contrast, the strong inhibition of STAT3 phosphorylation induced by NZ decreased the transcription of *IDO* mRNA, the release of kynurenine in the tumor supernatant and the infiltration of Treg cells.

The only decrease in Treg cells is sufficient to reverse the immunotolerant microenvironment created by solid tumors, also in the absence of changes in other classes of B- and T-lymphocytes [38, 53]. When effective, anthracycline-based regimens reduce the intra-tumor Foxp3A^+^ cells without changing the amount of CD8^+^ T-lymphocytes [54]. Interestingly, in a large phase III trial the decrease of intra-tumor Foxp3A^+^ cells has been predictive of anthracyclines efficacy in patients with breast cancer [55]. In JC resistant tumors, doxorubicin did not modify the percentage of intra-tumor Foxp3A^+^ Treg cells. This was likely an additional mechanism of doxorubicin resistance: of note, this feature was counteracted by the pre-treatment with NZ.

**Figure 8:**
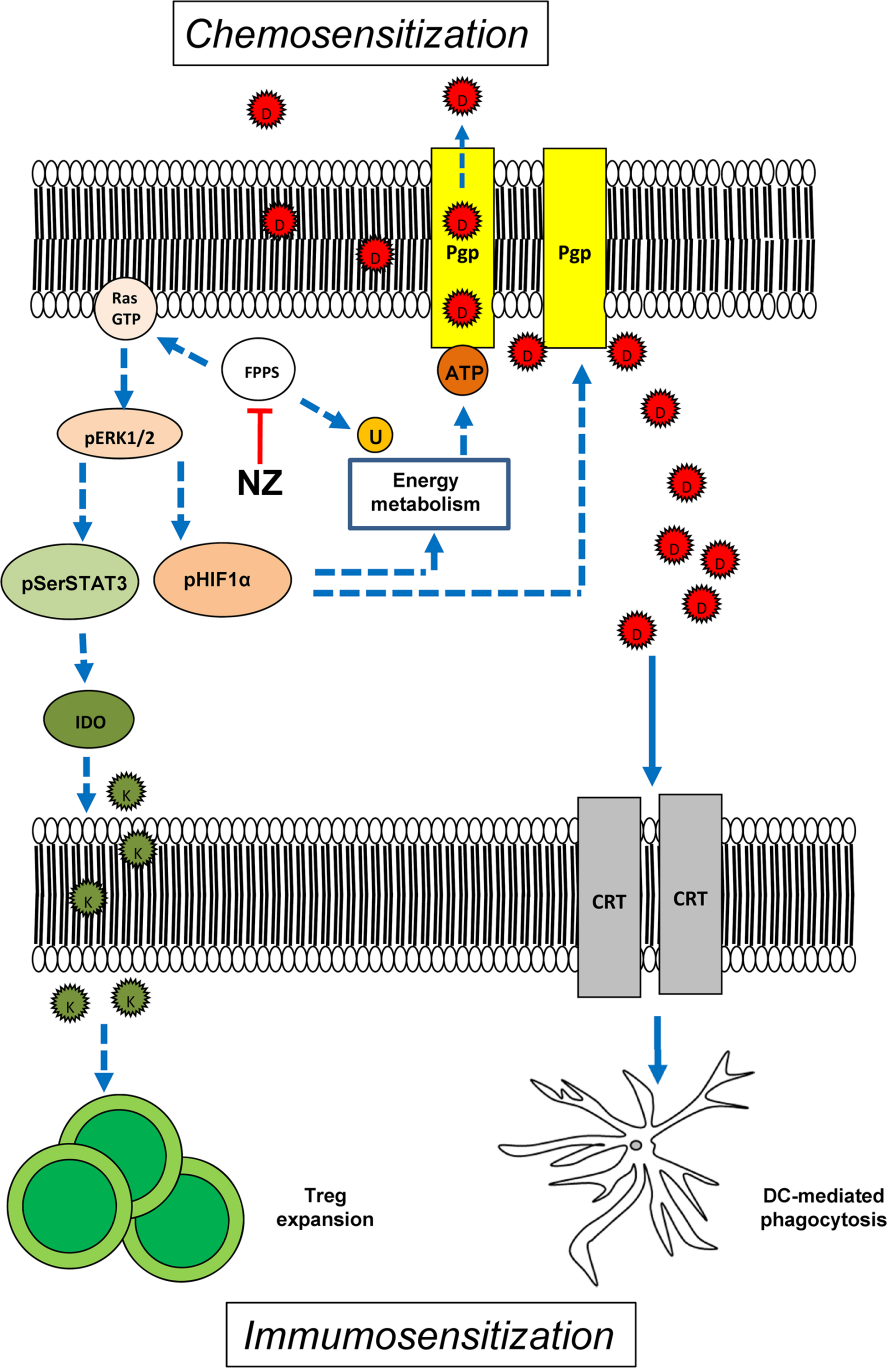
Effects of NZ on tumor cells and tumor-associated immune microenvironment By inhibiting the farnesyl pyrophosphate synthase (FPPS), self-assembling zoledronic acid formulation (NZ) reduces the isoprenoids supply. This event implies: 1) lower activity of Ras/ERK1/2/HIF-1α axis, lower HIF-1α-dependent transcription of P-glycoprotein (Pgp) and lower production of ATP via HIF-1α-dependent anaerobic energy metabolism; 2) reduced amount of the electron carrier ubiquinone (U), which decreases the mitochondrial aerobic energy metabolism and further diminishes the ATP supply for Pgp; 3) reduced activity of Ras/ERK1/2/STAT3 axis, lower expression of indoleamine 2,3 dioxygenase (IDO) and lower production of the immunosuppressive metabolite kynureine (K) that is required for the expansion of the tumor-tolerant T-regulatory (Treg) cells. The impairment in tumor energy metabolism and the reduction of Treg may explain the anti-tumor effect of NZ. When co-administered with doxorubicin (D), NZ reduces the drug efflux owing to the lower expression of Pgp; in parallel, it increases the doxorubicin-mediated exposure of calreticulin (CRT) that activates the tumor cell phagocytosis by local dendritic cells (DC). Hence, NZ induces chemosensitization and immunosensitization. Dotted lines: pathways inhibited by NZ; continuous lines: pathways activated by NZ.

In conclusion our work suggests that NZ is a multi-target chemo-immunosensitizing agent, acting on both tumor cell and tumor microenvironment. On the one hand, it exerts cytotoxic effects against breast tumors refractory to doxorubicin when used alone, by impairing the tumor energy metabolism and reducing the tumor-induced immunosuppression. On the other hand, NZ overcomes the resistance to doxorubicin by at least two mechanisms: 1) it increases the intracellular retention of the drug, enhancing the direct cytotoxicity of doxorubicin on tumor cells; 2) it restores the doxorubicin-induced immunogenic cell death, re-establishing an anti-tumor immune microenvironment (Figure 8). We propose the use of NZ as an adjuvant tool in chemo-immunotherapy protocols for the treatment of anthracycline-refractory breast tumors.

## MATERIALS AND METHODS

### Ethical statement

Investigation has been conducted in accordance with the ethical standards, the Declaration of Helsinki, national and international guidelines, and has been approved by the Bio-Ethical Committee of the University of Turin, Italy.

### Chemicals

Fetal bovine serum and culture medium were from Invitrogen Life Technologies (Carlsbad, CA). Plasticware for cell cultures was from Falcon (Becton Dickinson, Franklin Lakes, NJ). ZA was a gift from Novartis (Basel, Switzerland). Electrophoresis reagents were obtained from Bio-Rad Laboratories (Hercules, CA). The protein content in cell lysates and tumor homogenates was assessed with the BCA kit from Sigma Chemical Co. (St. Louis, MO). Unless otherwise specified, all the other reagents were purchased from Sigma Chemical Co.

### Preparation and characterization of NZ

Self-assembling NPs encapsulating ZA were prepared as previously reported [28]. Briefly, an aqueous solution of 18 mmol/L CaCl_2_ was added, dropwise and under magnetic stirring, to an aqueous solution of 10.8 mmol/L Na_2_HPO_4_. The resulting suspension (termed CaPNPs) was filtered through a 0.22 μm polycarbonate filter (MF-Millipore, Microglass Heim, Italy) and stored at 4°C before use. ZA was then complexed with CaPNPs (to obtain CaPZNPs), at a volume ratio of 50:1, with a final ZA concentration of 50 mg/mL. Cationic liposomes (N-[1-(2,3-dioleoyloxy)propyl]-N,N,N-trimethylammonium chloride/cholesterol/1,2-distearoyl-*sn*-glycero-3-phosphoethanolamine-N-[amino (polyethylene glycol)-2000] at a ratio of 1:1:0.5) were prepared by hydration of a thin lipid film followed by extrusion. The lipid mixture dissolved in chloroform/methanol (2:1 v/v) was added to a 50 mL round-bottom flask and the solvent was removed under reduced pressure by a rotary evaporator (Laborota 4010 digital, Heidolph, Schwabach, Germany) in nitrogen atmosphere. The resulting lipid film was hydrated with 1 mL of 0.22 μm-filtered distilled water and the resulting suspension was gently mixed in the presence of glass beads followed by incubation at room temperature for 2 h. The liposome suspension was then extruded using a thermobarrel extruder system (Northern Lipids Inc., Vancouver, BC, Canada) passing repeatedly the suspension under nitrogen atmosphere through polycarbonate membranes with decreasing pore sizes from 400 to 100 nm (Nucleopore Track Membrane 25 mm, Whatman, Brentford, UK). The liposomes were stored at 4°C. Each formulation was prepared in triplicate. Finally, equal volumes of suspensions of the liposomes and CaPZNPs, respectively, were mixed in a glass tube and the resulting dispersion was maintained at room temperature for 10 min. NPs without ZA were also prepared similarly, starting from CaPNPs and cationic liposomes. Each formulation was prepared in triplicate.

The mean diameter of stealth liposomes and CaPZNPs were determined at 20°C by photon correlation spectroscopy (N5, Beckman Coulter, Miami, FL). Each sample was diluted in deionizer/filtered water and analyzed with detector at 90° angle. Polydispersity index (P.I.) was used as measure of the particle size distribution. For each batch, the mean diameter and size distribution were the mean of three measures. For each formulation, the mean diameter and P.I. were calculated as the mean of three different batches. The zeta potential (ζ) of the NP surface was measured in water by means of a Zetasizer Nano Z (Malvern, UK). Data of ζ were collected as the average of 20 measurements. To measure the ZA encapsulation efficacy, 1 mL of NP dispersion was ultra-centrifuged (Optima Max E, Beckman Coulter) at 80,000 × g at 4°C for 40 min. Supernatants were carefully removed and ZA concentration was determined by high pressure liquid chromatography. The ZA encapsulation efficiency was calculated as [(TS_ZA_ – AS_ZA_)/TS_ZA_] × 100, where TS_ZA_ is the theoretical ZA in the supernatant and AS_ZA_ is the actual ZA concentration in the supernatant.

### Cells

Human non-transformed breast epithelial MCF10A cells, human breast cancer MCF7, SKBR3, T74D, MDA-MB-231 cells and murine mammary cancer JC cells were purchased from ATCC (Manassas, VA). Murine mammary cancer TUBO cells were a kind gift of Prof. Federica Cavallo, Department of Molecular Biotechnology and Health Sciences, University of Turin, Italy. Cells were maintained in medium supplemented with 10% v/v fetal bovine serum, 1% v/v penicillin-streptomycin, 1% v/v L-glutamine. 2 × 10^5^ JC cells were stably transfected with 2 μg of pGL4.51[luc2/CMV/Neo] vector (Promega Corporation, Madison, WI), then selected in culture medium containing 1 μg/mL neomycin to generate the stably luciferase-expressing JC-luc clone.

### Western blot analysis

Cells were lysed in MLB buffer (125 mmol/L Tris-HCl, 750 mmol/L NaCl, 1% v/v NP40, 10% v/v glycerol, 50 mmol/L MgCl_2_, 5 mmol/L EDTA, 25 mmol/L NaF, 1 mmol/L NaVO_4_, 10 μg/mL leupeptin, 10 μg/mL pepstatin, 10 μg/mL aprotinin, 1 mmol/L phenylmethylsulfonyl fluoride, pH 7.5), sonicated and centrifuged at 13,000 × g for 10 min at 4°C. Tumors were frozen at −80°C for 24 h, then homogenized in MLB buffer and treated as reported above. 20 μg of proteins from cell lysates or tumor homogenates were subjected to Western blotting and probed with the following antibodies: anti-Pgp (BD Biosciences, San Josè, CA), anti-MRP1 (Abcam, Cambridge, UK), anti-Ras (Millipore, Billerica, MA), anti-phospho(Thr202/Tyr204, Thr185/Tyr187)-ERK1/2 (Millipore), anti-ERK 1/2 (Millipore), anti-phospho(Tyr705)-STAT3 (Cell Signaling Technology, Danvers, MA), anti-phospho(Ser727)-STAT3 (Cell Signaling Technology), anti-STAT3 (Thermo Scientific, Rockford, IL). The proteins were detected by enhanced chemiluminescence (Bio-Rad Laboratories).

To evaluate the activity of Ras, the Ras GTP-bound fraction, taken as an index of the active protein, was measured in a pull-down assay using Raf-1-GST fusion protein-agarose beads-conjugates (Millipore) and then probing the immuno-precipitated samples with an anti-Ras (Millipore) antibody. Nuclei-cytosol separation was obtained with the Nuclear Extract Kit (Active Motif, Rixensart, Belgium). To assess HIF-1α phosphorylation, the cytosolic lysate was immunoprecipitated with a polyclonal anti-HIF-1α antibody (Santa Cruz Biotechnology Inc., Santa Cruz, CA), then resolved by SDS-PAGE and probed with a biotin-conjugated anti-phosphoserine antibody (Sigma Chemical Co.), followed by polymeric streptavidin-horseradish peroxidase-conjugates (Sigma Chemical Co.). 10 μg nuclear proteins were resolved by SDS-PAGE and probed with an anti-HIF-1α antibody (BD Biosciences), to measure the amount of protein translocated into the nucleus. The amount of cytochrome c in the cytosolic and mitochondrial extracts was measured as described in [56], using an anti-cytochrome c antibody (BD Biosciences). The equal protein loading in whole cell lysates, nuclear and mitochondrial extracts was checked by using anti-actin (Sigma Chemical Co.), anti-TATA-box binding protein (TBP; Santa Cruz Biotechnology Inc.), anti-porin (Abcam) antibodies, respectively.

### Intracellular doxorubicin accumulation

Cells were incubated for 3 h with 5 μmol/L doxorubicin, then washed twice in PBS, detached with trypsin-EDTA, centrifuged at 1,300 × g for 2 min, sonicated and re-suspended in 0.5 mL ethanol/0.3 N HCl. The amount of doxorubicin was measured spectrofluorimetrically, using a Synergy HT Multi-Detection Microplate Reader (Bio-Tek Instruments, Winooski, VT). Excitation and emission wavelengths were 475 nm and 553 nm. Fluorescence was converted into nmoles/mg cell proteins, using a calibration curve previously set.

### Cell viability

To determine IC_50_, 5 × 10^5^ cells were incubated in the absence or presence of 1 μmol/L ZA or NZ for 72 h, with increasing concentrations of doxorubicin (from 1 nmol/L to 1 mmol/L) in the last 48 h. IC_50_ was considered the concentration of doxorubicin that kills 50% cells. Viable cells were quantified by the neutral red staining method, as previously reported [57]: at the end of the treatment period, cells were incubated for 1 h at 37°C in culture medium containing 70 μg/mL of neutral red solution (Sigma Chemical Co.), then washed three times with PBS and rinsed with stop buffer (32 mmol/L trisodium citrate, 50% v/v methanol, pH 6). The absorbance at 540 nm was read using a Synergy HT Multi-Detection Microplate Reader (Bio-Tek Instruments). The absorbance of untreated cells was considered as 100% viability; the results were expressed as percentage of viable cells versus untreated cells.

### Cytotoxicity assays

The release of HMGB1 in the cell culture supernatant was measured using the High Mobility Group Protein 1 ELISA kit (Cloud-Clone Corp., Houston, Texas), following the manufacturer's instructions. Results were expressed in pg/mg cell proteins, according to a titration curve previously prepared. In parallel, 20 μL of the cell culture medium were resolved by SDS-PAGE and probed with an anti-HMGB1 antibody (Sigma Chemical Co.). Blots were pre-stained with Red Ponceau, to check the equal loading of proteins.

### *In vivo* assays

1 × 10^6^ JC or JC-luc cells, mixed with 100 μL Matrigel, were injected subcutaneously in 6 weeks-old immunocompetent or NOD SCID female BALB/c mice, housed (5 per cages) under 12 h light/dark cycle, with food and drinking provided *ad libitum*. Palpable tumors developed 2-3 weeks after injection. The tumor growth was measured daily by caliper and was calculated according to the equation (L × W^2^)/2, where L = tumor length; W = tumor width. When the tumor reached the volume of 60 mm^3^, the mice were randomized into 6 groups: 1) Ctrl group, treated with 0.1 mL saline solution i.v. at day 3, 9, 15; 2) Dox group, treated with 5 mg/kg doxorubicin i.v. at day 3, 9, 15; 3) ZA group, treated with 20 μg/mouse ZA i.v. at day 2, 8, 14; 4) ZA + Dox group, treated with 20 μg/mouse ZA i.v. at day 2, 8, 14 followed by 5 mg/kg doxorubicin i.v. at day 3, 9, 15; 5) NZ group, treated with 20 μg/mouse NZ i.v. at day 2, 8, 14; 6) NZ + Dox group, treated with 20 μg/mouse NZ i.v. at day 2, 8, 14 followed by 5 mg/kg doxorubicin i.v. at day 3, 9, 15. *In vivo* bioluminescence imaging was performed at day 0, 6, 12, 18 after randomization, with a Xenogen IVIS Spectrum (PerkinElmer, Waltham, MA). Tumor volumes were monitored daily and animals were euthanized by injecting i.m. zolazepam (0.2 mL/kg) and xylazine (16 mg/kg) at day 21 after randomization. The hematochemical parameters LDH, AST, ALT, AP, CPK, creatinine were measured on 0.5 mL of blood collected immediately after mice sacrifice, using commercially available kits from Beckman Coulter Inc.

### Immunohistochemistry

Tumors were resected, photographed and fixed in 4% v/v paraformaldehyde. The sections were stained with hematoxylin/eosin or immunostained for Ki67 (Millipore), Pgp (Millipore), CRT (Affinity Bioreagents, Golden, CO), CD11c (BD Biosciences), CD19 (BD Biosciences), CD4 (Abcam), CD8 (Abcam), Foxp3A (eBioscience, San Diego, CA), followed by a peroxidase-conjugated secondary antibody (Dako, Glostrup, Denmark). Nuclei were counterstained with hematoxylin.

### HIF-1α activity

The activity of HIF-1α was assessed on 10 μg proteins from nuclear extracts with the TransAM^™^ HIF-1 Transcription Factor Assay Kit (Active Motif). Data were expressed as mU absorbance/mg nuclear proteins. For each set of experiments, a blank (with bis-distilled water), a negative control (with mutated oligonucleotide) and a competition assay (with 10 pmoles of the wild type oligonucleotide and 100 pmoles of the mutated oligonucleotide incubated with the nuclear extracts of JC cells grown at 3% O_2_ for 24 h), were included. In hypoxic conditions, the activity of HIF-1α was 307.23 ± 12.81 mU/mg nuclear proteins; in the competition assay, the corresponding HIF-1α activity was reduced to 27.75 ± 5.71 mU/mg nuclear proteins (*n* = 3; *p* < 0.001).

### Quantitative real time PCR (qRT-PCR)

Total RNA was extracted by phenol/chloroform method; 1 μg RNA was reverse-transcribed using the *iScript* Reverse Transcription Supermix kit (Bio-Rad Laboratories), according to the manufacturer's instruction. 25 ng cDNA were amplified with 10 μL IQ^™^ SYBR Green Supermix (Bio-Rad Laboratories), using 400 nmol/L of forward and reverse primers. Primers sequence is listed in the Supplementary Table 1. RT-PCR was carried out with a iQ^™^5 cycler (Bio-Rad Laboratories). Cycling conditions were: 30 s at 95°C, followed by 40 cycles of denaturation (15 s at 95°C), annealing/extension (30 s at 60°C). The same cDNA preparation was used to quantify the genes of interest and the housekeeping gene *S14*, used to normalize gene expression. The relative quantitation of each sample was performed using the Gene Expression Quantitation software (Bio-Rad Laboratories). Results were expressed in arbitrary units. For each gene, the expression in untreated cells was considered “1”.

### Glucose uptake and metabolism

The uptake of glucose was measured as described earlier [58], by radiolabeling cells with 0.3 μCi/mL 2-deoxy-D-[^3^H]-glucose (7.5 Ci/mmol, PerkinElmer). The uptake values were corrected for the non carrier-mediated transport by measuring the uptake in the presence of 10 μmol/L cytochalasin B, an inhibitor of the facilitated glucose transport: such value was always < 10% of the total uptake. The results were expressed as pmoles 2-deoxy-D-[^3^H]-glucose/mg cell proteins. The glucose flux through glycolysis and TCA cycle was measured by radiolabeling cells with 2 μCi/mL [6–^14^C]-glucose (55 mCi/mmol; PerkinElmer). Cell suspensions were incubated for 1 h at 37°C in a closed experimental system to trap the ^14^CO_2_ developed from [^14^C]-glucose, and the reaction was stopped by injecting 0.5 mL of 0.8 N HClO_4_. The amount of glucose transformed into CO_2_ through the glycolysis and the TCA cycle was calculated as described [59] and expressed as pmoles CO_2_/h/mg cell proteins.

### Mitochondrial respiratory chain assays

Mitochondria were extracted as described earlier [56]. A 50 μL aliquot was sonicated and used for the measurement of protein content or Western blotting; the remaining part was stored at −80°C until the use. To confirm the presence of mitochondrial proteins in the extracts, 10 μg of each sonicated sample was subjected to SDS-PAGE and probed with an anti-porin antibody (Abcam; data not shown). The electron flux from Complex I to Complex III was measured on 10 μg of non-sonicated mitochondrial extracts, re-suspended in 150 μL buffer A (5 mmol/L KH_2_PO_4_, 5 mmol/L MgCl_2_, 5% w/v bovine serum albumin). Then 75 μL buffer B (25% w/v saponin, 50 mmol/L KH_2_PO_4_, 5 mmol/L MgCl_2_, 5% w/v bovine serum albumin, 0.12 mmol/L cytochrome c-oxidized form, 0.2 mmol/L NaN_3_) were added for 5 min at room temperature. Each sample was incubated in the absence or presence of the Complex I inhibitor rotenone (50 μmol/L), to measure the ubiquinone-independent and the ubiquinone-dependent electron flux, respectively. The reaction was started with 0.15 mmol/L NADH and was followed for 5 min, using a Synergy HT Multi-Detection Microplate Reader (Bio-Tek Instruments). Results were expressed as nmoles reduced cytochrome c/min/mg mitochondrial proteins.

### ATP measurement

The amount of ATP was measured on 20 μg of mitochondrial proteins or on 100 μL of cell supernatants with the ATP Bioluminescent Assay Kit (FL-AA, Sigma Chemical Co.), using a Synergy HT Multi-Detection Microplate Reader (Bio-Tek Instruments). ATP was quantified as arbitrary light units; data were converted into nmoles/mg mitochondrial or cellular proteins, using a calibration curve previously set.

### ROS measurement

Mitochondria were extracted, re-suspended in a final volume of 0.5 mL PBS, incubated for 30 min at 37°C with the fluorescent probe 5-(and-6)-chloromethyl-2′,7′-dichlorodihydro-fluorescein diacetate-acetoxymethyl ester (5 μmol/L; DCFDA-AM), centrifuged at 13,000 × g for 5 min and re-suspended in 0.5 mL PBS. The fluorescence of each sample, taken as index of ROS level, was read at 492 nm (excitation) and 517 nm (emission), using a Synergy HT Multi-Detection Microplate Reader (Bio-Tek Instruments). The results were expressed as nmoles/mg mitochondrial proteins.

### Mitochondrial electric potential (Δψ) measurement

1 × 10^6^ cells re-suspended in 0.5 mL PBS were incubated for 30 min at 37°C with the fluorescent probe JC-1 (2 μmol/L; Biotium Inc., Hayward, CA), then centrifuged at 13,000 × g for 5 min and re-suspended in 0.5 mL PBS. The fluorescence of each sample was read using a Synergy HT Multi-Detection Microplate Reader (Bio-Tek Instruments): the red fluorescence, index of polarized mitochondria, was detected at 550 nm (excitation) and 600 nm (emission); the green fluorescence, index of depolarized and damaged mitochondria, was detected at 485 nm (excitation) and 535 nm (emission). The fluorescence units were used to calculate the percentage of green-fluorescent mitochondria versus red-fluorescent mitochondria.

### Caspases activity

The activity of caspase 9 and caspase 3 was measured as reported in [60]. Cells were lysed in 0.5 mL of caspase lysis buffer (20 mmol/L Hepes/KOH, 10 mmol/L KCl, 1.5 mmol/L MgCl_2_, 1 mmol/L EGTA, 1 mmol/L EDTA, 1 mmol/L dithiothreitol, 1 mmol/L phenylmethylsulfonyl fluoride, 10 μg/mL leupeptin, pH 7.5). 20 μg cell lysates were incubated for 1 h at 37°C with 20 μmol/L of the fluorogenic substrate of caspase 9 Ac-Leu-Glu-His-Asp-7-amino-4-methylcoumarin (LEHD-AMC) or fluorogenic substrate of caspase 3 Ac-Asp-Glu-Val-Asp-7-amino-4-methylcoumarin (DEVD-AMC), in 0.25 mL caspase assay buffer (25 mmol/L Hepes, 0.1% w/v 3-[(3-cholamidopropyl)dimethylammonio]-1-propanesulfonate, 10% w/v sucrose, 10 mmol/L dithiothreitol, 0.01% w/v egg albumin, pH 7.5). The reaction was stopped by adding 0.75 mL ice-cold 0.1% w/v trichloroacetic acid and the fluorescence of AMC fragment released by active caspases was read using a Synergy HT Multi-Detection Microplate Reader (Bio-Tek Instruments). Excitation and emission wavelengths were 380 and 460 nm, respectively. Fluorescence was converted in nmoles/mg cell proteins, using a calibration curve prepared previously with standard solutions of AMC.

### Flow cytometry analysis

1 × 10^6^ cells, rinsed and fixed with 2% w/v paraformaldehyde for 2 min, were washed three times with PBS and stained with an anti-CRT antibody (Affinity Bioreagents) for 1 h on ice. After washing, samples were incubated with an AlexaFluor 488-conjugated secondary antibody (Millipore) for 30 min and re-washed. Samples were analyzed with a Guava easyCyte flow cytometer (Millipore). For each analysis 10,000 events were collected. Control experiments included incubation with non immune isotypic antibody followed by the secondary antibody. The results were analyzed with the easyCyte software (Millipore).

### Kynurenine production

Tumor pieces were collected immediately after mice sacrifice and maintained for 24 h in culture medium, then 200 μL of the supernatants were used to measure the levels of kynurenine, taken as index of IDO activity, as previously detailed [17]. Supernatants were added to 100 μL of 30% w/v trichloroacetic acid and incubated for 30 min at 50°C to hydrolyze N-formylkynurenine to kynurenine. After centrifugation at 10,000 × g for 10 min, 100 μL of the supernatant were transferred into a 96-well plate, mixed with 100 μL of 2% w/v p-dimethylamino benzaldehyde in 99.8% v/v acetic acid, and incubated for 10 min at room temperature. Kynurenine was detected by measuring the absorbance at 490 nm, using a Synergy HT Multi-Detection Microplate Reader (Bio-Tek Instruments). The absorbance of the culture medium alone was considered as a blank and was subtracted from the values obtained in the presence of the cells. The results were expressed as nmol kynurenine/mg cell proteins, according to a titration curve previously set.

### FPP and ubiquinone synthesis

Cells were labeled with 1 μCi/mL [^3^H]-acetate (3600 mCi/mmol; Amersham Bioscience, Little Chalfont, UK). The synthesis of radiolabeled FPP and ubiquinone was measured as described in [61]. Results were expressed as pmoles [^3^H]-FPP or [^3^H]-ubiquinone/mg cell proteins, according to the relative calibration curve.

### Statistical analysis

All data in text and figures are provided as means ± SD. The results were analyzed by a one-way analysis of variance (ANOVA) and Tukey's test. *p* < 0.05 was considered significant.

## SUPPLEMENTARY MATERIALS FIGURES AND TABLE


