# Supplementary material for: Zoledronic acid-encapsulating self-assembling nanoparticles and doxorubicin: a combinatorial approach to overcome simultaneously chemoresistance and immunoresistance in breast tumors
Source: Oncotarget. 2016 Mar 9;7(15):20753–72. doi: 10.18632/oncotarget.8012 (PMC4991490; doi:10.18632/oncotarget.8012)
Supplement: Supplementary file 1 [file oncotarget-07-20753-s001.pdf]

Zoledronic acid-encapsulating self-assembling nanoparticles and doxorubicin: a combinatorial approach to overcome simultaneously chemoresistance and immunoresistance in breast tumors

Supplementary Materials

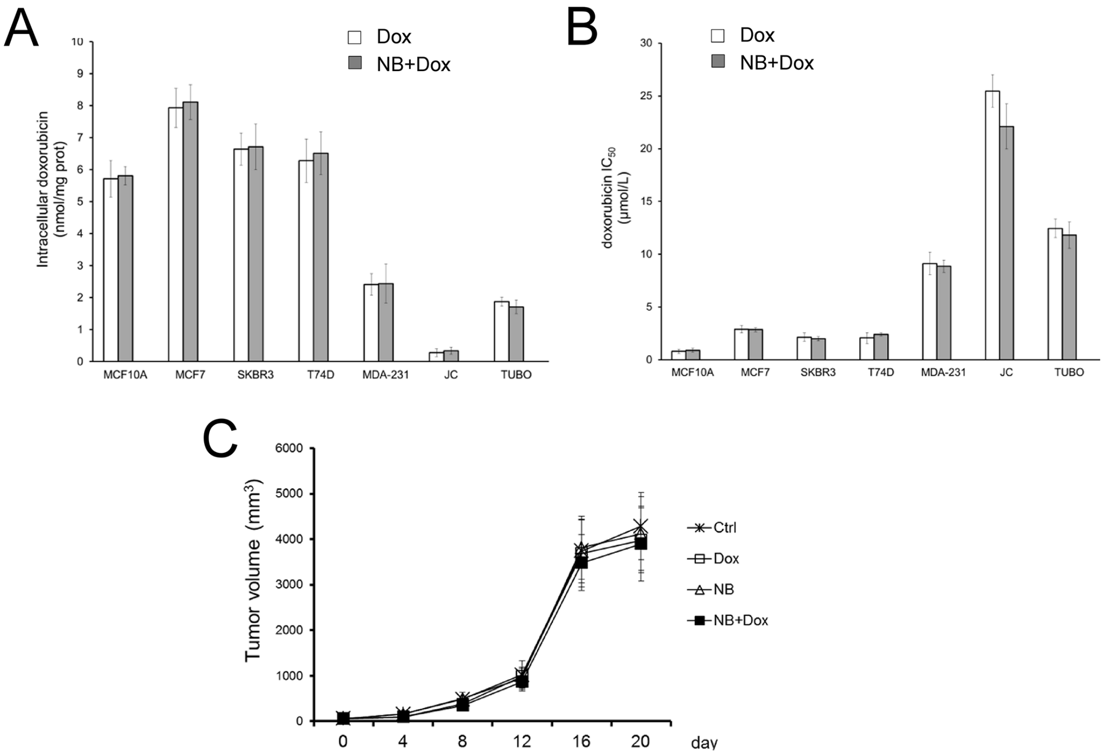

Supplementary Figure S1: Nanoparticles without zoledronic acid do not reverse doxorubicin resistance. Human non transformed breast epithelial MCF10A cells, human breast cancer MCF7, SKBR3, T74D, MDA-MB-231 cells, murine mammary cancer TUBO and JC cells were subjected to the following investigations. (**A**) Cells were incubated for 24 h with 5 µmol/L doxorubicin (Dox) or with 1 µmol/L nanoparticles without zoledronic acid (blank nanoparticles, NB) for 24 h, followed by 5 µmol/L doxorubicin for additional 24 h (NB + Dox). The intracellular content of doxorubicin was measured spectrofluorimetrically in duplicate (n = 4). Data are presented as means ± SD. There were not statistically significant differences between Dox and NB + Dox in each cell line. (**B**) Cells were left untreated or incubated for 72 h in the presence of 1 µmol/L NB; different concentrations (1 nmol/L, 10 nmol/L, 100 nmol/L, 1 µmol/L, 10 µmol/L, 100 µmol/L, 1 mmol/L) of doxorubicin (Dox) were added in the last 48 h. Samples were then stained in quadruplicate with the neutral red solution ($n = 4$). IC_{50} was calculated as the concentration of doxorubicin that kills 50% of cells. Data are presented as means ± SD. There were not statistically significant differences between Dox and NB + Dox in each cell line. (**C**) Six weeks-old female BALB/c mice bearing 60 mm^3 JC tumors were randomly divided into the following groups (10 mice/group): 1) Ctrl group, treated with 0.1 mL saline solution i.v. at day 3, 9, 15; 2) Dox group, treated with 5 mg/kg doxorubicin i.v. at day 3, 9, 15; 3) NB group, treated with 20 µg/mouse nanoparticle formulation without zoledronic acid i.v. at day 2, 8, 14; 4) NB + Dox group, treated with 20 µg/mouse NB i.v. at day 2, 8, 14 followed by 5 mg/kg doxorubicin i.v. at day 3, 9, 15. Tumor growth was monitored daily by caliper measurement. Data are presented as means ± SD. There were not statistically significant differences among each group of treatment.

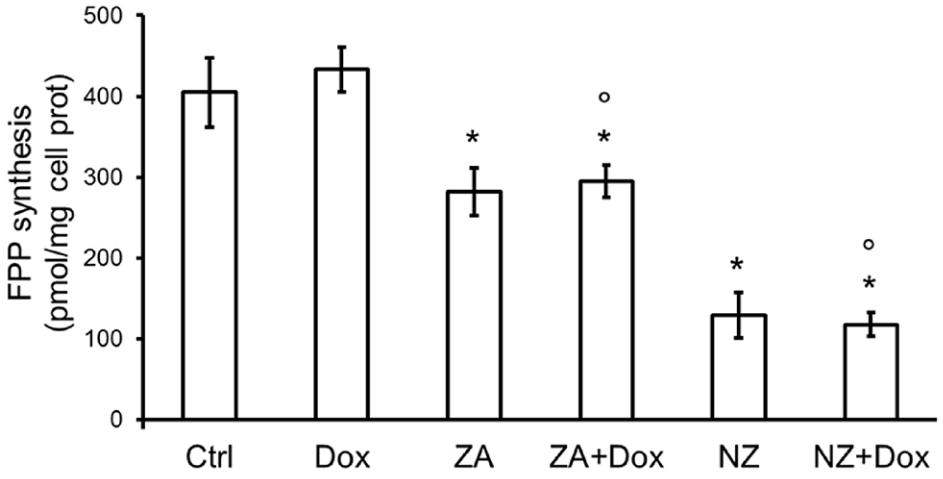

Supplementary Figure S2: NZ lowers the synthesis of FPP in chemoresistant cells. JC cells were grown in fresh medium (Ctrl) or medium containing 5 µmol/L doxorubicin (Dox, 24 h), 1 µmol/L zoledronic acid (ZA, 48 h), 1 µmol/L ZA for 24 h followed by 5 µmol/L doxorubicin for additional 24 h (ZA + Dox), 1 µmol/L self-assembling ZA formulation (NZ, 48 h), 1 µmol/L NZ for 24 h followed by 5 µmol/L doxorubicin for additional 24 h (NZ + Dox). Cells were radiolabeled during the last 24 h with [^{3}H]-acetate, then the *de novo* synthesis of FPP was measured. Data are presented as means ± SD ($n = 3$). Versus Ctrl: *$p < 0.05$; versus Dox: °$p < 0.005$.

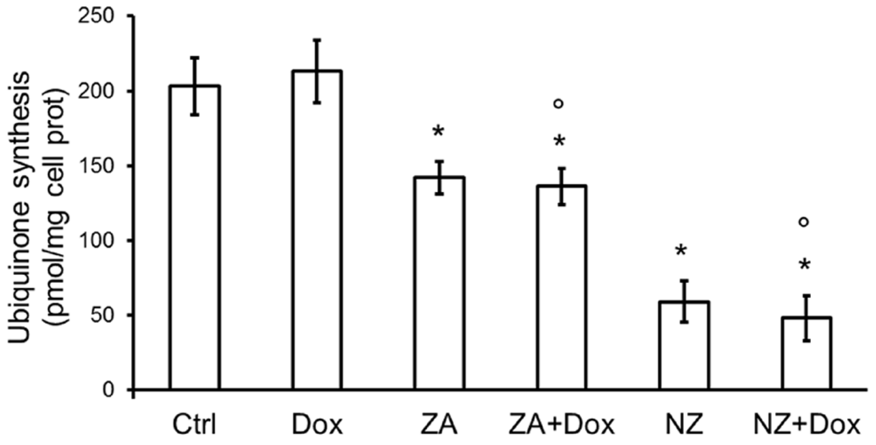

Supplementary Figure S3: NZ lowers the synthesis of ubiquinone in chemoresistant cells. JC cells were grown in fresh medium (Ctrl) or medium containing 5 µmol/L doxorubicin (Dox, 24 h), 1 µmol/L zoledronic acid (ZA, 48 h), 1 µmol/L ZA for 24 h followed by 5 µmol/L doxorubicin for additional 24 h (ZA + Dox), 1 µmol/L self-assembling ZA formulation (NZ, 48 h), 1 µmol/L NZ for 24 h followed by 5 µmol/L doxorubicin for additional 24 h (NZ + Dox). Cells were radiolabeled during the last 24 h with [^{3}H]-acetate, then the *de novo* synthesis of ubiquinone was measured. Data are presented as means ± SD ($n = 3$). Versus Ctrl: *$p < 0.05$; versus Dox: °$p < 0.001$.

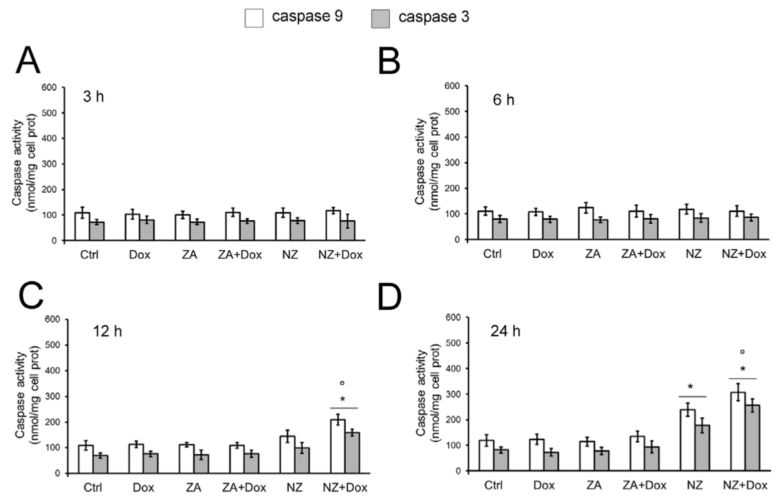

Supplementary Figure S4: Time-dependent activation of caspase 9 and caspase 3 in chemoresistant cells. (**A–D**) JC cells were grown in fresh medium (Ctrl) or medium containing 5 µmol/L doxorubicin (Dox, 24 h), 1 µmol/L zoledronic acid (ZA, for 3, 6, 12, 24 h as indicated in each panel), 1 µmol/L ZA for the indicated periods followed by 5 µmol/L doxorubicin for additional 24 h (ZA + Dox), 1 µmol/L self-assembling ZA formulation (NZ, for 3, 6, 12, 24 h as indicated in each panel), 1 µmol/L NZ for the indicated periods followed by 5 µmol/L doxorubicin for additional 24 h (NZ + Dox). The activities of caspase 9 and 3 were measured spectrofluorimetrically in the cell lysates. Data are presented as means ± SD ($n = 3$). Versus Ctrl: *$p < 0.01$; versus Dox: °$p < 0.005$.

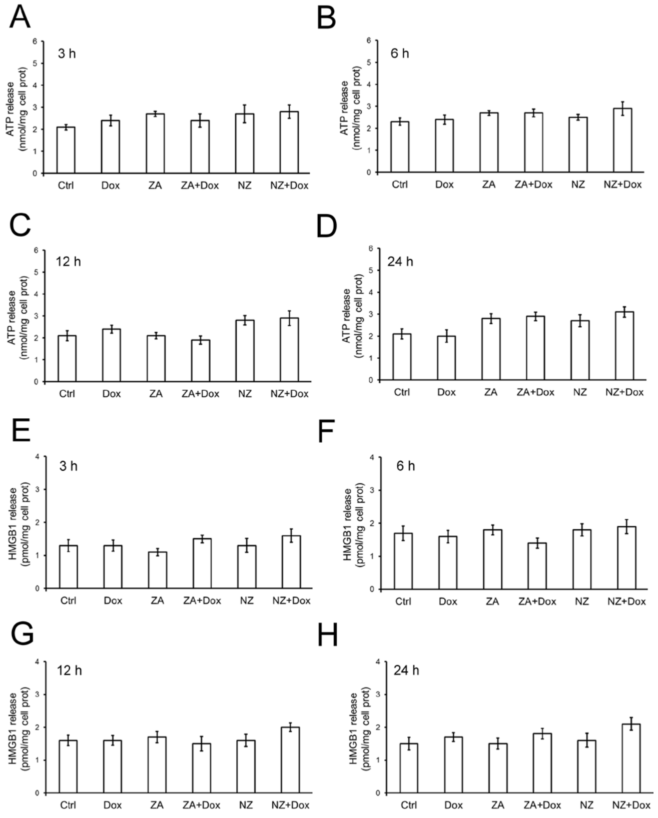

Supplementary Figure S5: Time-dependent release of ATP and HMGB1 in chemoresistant cells. JC cells were grown in fresh medium (Ctrl) or medium containing 5 µmol/L doxorubicin (Dox, 24 h), 1 µmol/L zoledronic acid (ZA, for 3, 6, 12, 24 h as indicated in each panel), 1 µmol/L ZA for the indicated periods followed by 5 µmol/L doxorubicin for additional 24 h (ZA + Dox), 1 µmol/L self-assembling ZA formulation (NZ, for 3, 6, 12, 24 h as indicated in each panel), 1 µmol/L NZ for the indicated periods followed by 5 µmol/L doxorubicin for additional 24 h (NZ + Dox). (**A–D**) The extracellular release of ATP was measured by a chemiluminescence-based assay. Data are presented as means ± SD ($n = 3$). There were not statistically significant differences among each group of treatment. (**E–H**) The extracellular release of HMGB1 was measured by ELISA. Data are presented as means ± SD ($n = 3$). There were not statistically significant differences among each group of treatment.

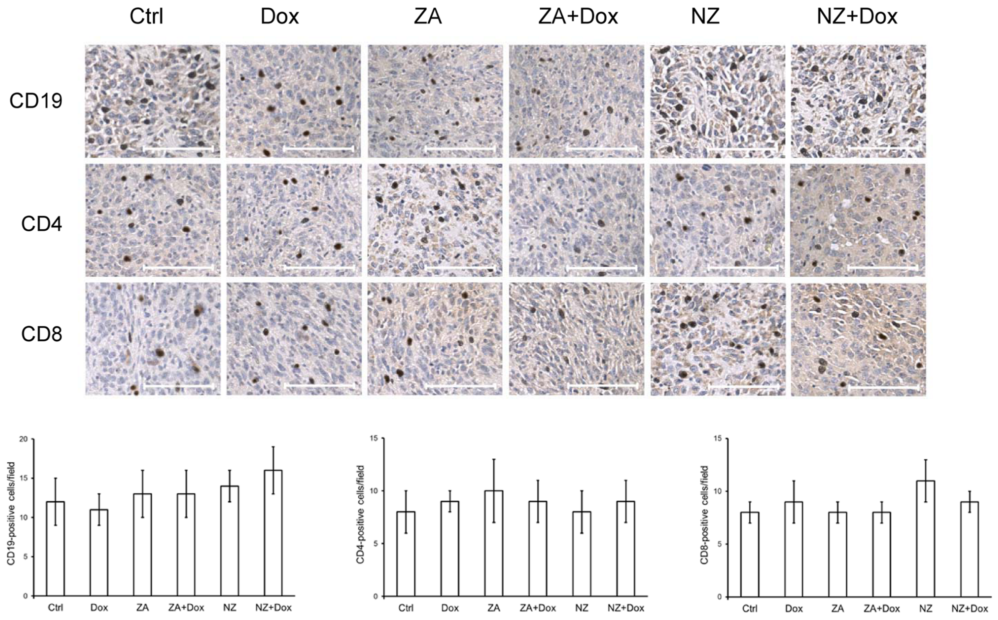

Supplementary Figure S6: Histochemical analysis of immune cells infiltrating mammary chemoresistant JC tumors. Six weeks-old female BALB/c mice bearing 60 mm^3 JC-luc tumors were randomly divided into the following groups (10 mice/group): 1) Ctrl group, treated with 0.1 mL saline solution i.v. at day 3, 9, 15; 2) Dox group, treated with 5 mg/kg doxorubicin i.v. at day 3, 9, 15; 3) ZA group, treated with 20 µg/mouse ZA i.v. at day 2, 8, 14; 4) ZA + Dox group, treated with 20 µg/mouse ZA i.v. at day 2, 8, 14 followed by 5 mg/kg doxorubicin i.v. at day 3, 9, 15; 5) NZ group, treated with 20 µg/mouse self-assembling ZA formulation i.v. at day 2, 8, 14; 6) NZ + Dox group, treated with 20 µg/mouse NZ i.v. at day 2, 8, 14 followed by 5 mg/kg doxorubicin i.v. at day 3, 9, 15. Sections of tumors from each group of animals were immunostained for CD19, a marker of B-lymphocytes; CD4, a marker of T-helper lymphocytes; CD8, a marker of T-cytotoxic lymphocytes. Nuclei were counter-stained with hematoxylin. Bar = 10 µm (63× objective). The photographs are representative of sections from 10 tumors/group. The number of positive cells/field was calculated by analyzing sections from 10 animals of each group (111–75 cells/field), using ImageJ software (http://imagej.nih.gov/ij/). Data are presented as means ± SD. There were not statistically significant differences among each group of treatment.

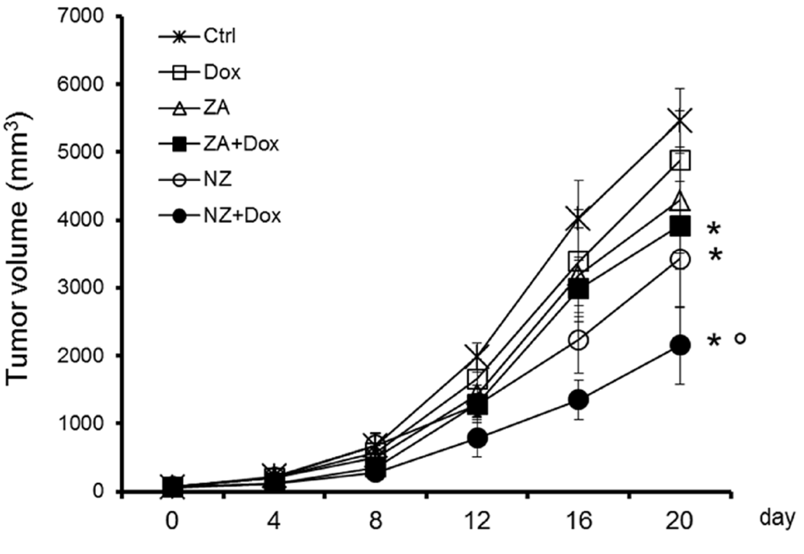

Supplementary Figure S7: Anti-tumor effects of NZ in immunodeficient mice. Six weeks-old female NOD SCID BALB/c mice bearing 60 mm^3 JC tumors were randomly divided into the following groups (10 mice/group): 1) Ctrl group, treated with 0.1 mL saline solution i.v. at day 3, 9, 15; 2) Dox group, treated with 5 mg/kg doxorubicin i.v. at day 3, 9, 15; 3) ZA group, treated with 20 µg/mouse ZA i.v. at day 2, 8, 14; 4) ZA + Dox group, treated with 20 µg/mouse ZA i.v. at day 2, 8, 14 followed by 5 mg/kg doxorubicin i.v. at day 3, 9, 15; 5) NZ group, treated with 20 µg/mouse self-assembling ZA formulation i.v. at day 2, 8, 14; 6) NZ + Dox group, treated with 20 µg/mouse NZ i.v. at day 2, 8, 14 followed by 5 mg/kg doxorubicin i.v. at day 3, 9, 15. Tumor growth was monitored daily by caliper measurement. Data are presented as means ± SD. Versus Ctrl group: *$p < 0.01$; NZ + Dox group in NOD SCID mice versus NZ + Dox group in immunocompetent mice (Figure 2C): °$p < 0.02$.

Supplementary Table S1: Primers sequence for qRT-PCR

Gene	Forward primer	Reverse primer
GLUT1	CCTGCAGTTTGGCTACAACA	TAACGAAAAGGCCCACAGAG
HK	AGACGCACCCACAGTATTCC	CGCATCCTCTTCTTCACCTC
PFK1	GGAGCTTCGAGAACAACTGG	CTGTGTGTCCATGGGAGATG
GAPDH	GAAGGTGAAGGTCGGAGT	CATGGTGGAATCATATTGGAA
ENO-A	GCTCCGGGACAATGATAAGA	TCCATCCATCTCGATCATCA
PK	TGCAGTGGAGCTCAGAGAGA	GCTTCCGGTGACATAATGCT
Pgp	TGCTGGAGCGGTTCTACG	ATAGGCAATGTTCTCAGCAATG
IDO	CAGGCAGATGTTTAGCAATGA	GATGAAGAAGTGGGCTTTGC
S14	GGTGCAAGGAGCTGGGTAT	TCCAGGGGTCTTGGTCCTATTT
